# *Anopheles stephensi* p38 MAPK signaling regulates innate immunity and bioenergetics during *Plasmodium falciparum* infection

**DOI:** 10.1186/s13071-015-1016-x

**Published:** 2015-08-19

**Authors:** Bo Wang, Nazzy Pakpour, Eleonora Napoli, Anna Drexler, Elizabeth K. K. Glennon, Win Surachetpong, Kong Cheung, Alejandro Aguirre, John M. Klyver, Edwin E. Lewis, Richard Eigenheer, Brett S. Phinney, Cecilia Giulivi, Shirley Luckhart

**Affiliations:** Department of Medical Microbiology and Immunology, School of Medicine, University of California Davis, 3437 Tupper Hall, One Shields Avenue, Davis, CA 95616 USA; Department of Molecular Biosciences, School of Veterinary Medicine, University of California Davis, Davis, CA USA; Department of Entomology and Nematology, University of California Davis, Davis, CA USA; Genome and Biomedical Sciences Center, University of California Davis, Davis, CA USA; Medical Investigations of Neurodevelopmental Disorders (MIND) Institute, University of California Davis, Davis, CA USA

**Keywords:** Malaria, Mitochondria, Innate immunity, Mitogen-activated protein kinase, MAPK, p38, *Plasmodium*, *Anopheles*, OXPHOS

## Abstract

**Background:**

Fruit flies and mammals protect themselves against infection by mounting immune and metabolic responses that must be balanced against the metabolic needs of the pathogens. In this context, p38 mitogen-activated protein kinase (MAPK)-dependent signaling is critical to regulating both innate immunity and metabolism during infection. Accordingly, we asked to what extent the Asian malaria mosquito *Anopheles stephensi* utilizes p38 MAPK signaling during infection with the human malaria parasite *Plasmodium falciparum*.

**Methods:**

*A. stephensi* p38 MAPK (AsP38 MAPK) was identified and patterns of signaling *in vitro* and *in vivo* (midgut) were analyzed using phospho-specific antibodies and small molecule inhibitors. Functional effects of AsP38 MAPK inhibition were assessed using *P. falciparum* infection, quantitative real-time PCR, assays for reactive oxygen species and survivorship under oxidative stress, proteomics, and biochemical analyses.

**Results:**

The genome of *A. stephensi* encodes a single p38 MAPK that is activated in the midgut in response to parasite infection. Inhibition of A*s*P38 MAPK signaling significantly reduced *P. falciparum* sporogonic development. This phenotype was associated with A*s*P38 MAPK regulation of mitochondrial physiology and stress responses in the midgut epithelium, a tissue critical for parasite development. Specifically, inhibition of A*s*P38 MAPK resulted in reduction in mosquito protein synthesis machinery, a shift in glucose metabolism, reduced mitochondrial metabolism, enhanced production of mitochondrial reactive oxygen species, induction of an array of anti-parasite effector genes, and decreased resistance to oxidative stress-mediated damage. Hence, *P. falciparum*-induced activation of A*s*P38 MAPK in the midgut facilitates parasite infection through a combination of reduced anti-parasite immune defenses and enhanced host protein synthesis and bioenergetics to minimize the impact of infection on the host and to maximize parasite survival, and ultimately, transmission.

**Conclusions:**

These observations suggest that, as in mammals, innate immunity and mitochondrial responses are integrated in mosquitoes and that A*s*P38 MAPK-dependent signaling facilitates mosquito survival during parasite infection, a fact that may attest to the relatively longer evolutionary relationship of these parasites with their invertebrate compared to their vertebrate hosts. On a practical level, improved understanding of the balances and trade-offs between resistance and metabolism could be leveraged to generate fit, resistant mosquitoes for malaria control.

**Electronic supplementary material:**

The online version of this article (doi:10.1186/s13071-015-1016-x) contains supplementary material, which is available to authorized users.

## Background

The biological complexity of the malaria parasite life cycle has made it an elusive target for drug and vaccine development. This has led to the re-emergence and further spread of this disease, with over 250 million new cases of malaria occurring annually [1]. The malaria parasite life cycle involves development and transmission by *Anopheles* mosquitoes. *Plasmodium* development in *Anopheles* begins with ingestion by the mosquito of blood containing male and female gametocytes; these fuse within minutes of ingestion to form mobile ookinetes that penetrate the midgut epithelium 24-36 h later and rapidly transform into vegetative oocysts. After growth and development for 10-12 days (the extrinsic incubation period or EIP), hundreds to thousands of sporozoites are released from each oocyst into the hemolymph, the open circulatory system of the mosquito. These sporozoites invade the salivary glands, where they are released into the saliva and injected into a human host during subsequent blood feeding. As parasites undergo these developmental transitions, they are simultaneously exposed to physical, chemical, and immune barriers of the mosquito that act to limit infection [2, 3]. Chemical barriers of the midgut, such as increased reactive nitrogen and oxygen species (RNOS) can kill parasites directly in the gut lumen, preventing contact between parasites and the epithelium [4–9]. Additional immune defenses are activated when parasites encounter the midgut epithelium and include the production of immune effectors such as TEP1 [10, 11], APL1 [12, 11, 13], LRIM1 [14, 11], and LRRD7 [15], which together function to further limit parasite development. For successful infection, ookinetes must traverse the midgut by migration through or between midgut epithelial cells [16]; therefore, the integrity of the physical barrier of the midgut epithelial can also limit parasite development [7].

Mitogen-activated protein kinases (MAPKs) are serine/threonine protein kinases that regulate a variety of cellular processes. The three main families of MAPKs are the extracellular signal-regulated kinases (ERKs), c-Jun N-terminal kinases (JNKs) and p38 MAPKs (reviewed in [17]). P38 MAPK is highly conserved and critical to innate immune responses in a variety of organisms [18–21]. In mammals, four p38 MAPK isoforms can be activated by bacterial, viral, and parasitic stimuli (reviewed in [22]). *Caenorhabditis elegans* has a single p38 MAPK ortholog (PMK-1) that regulates antimicrobial peptide expression (reviewed in [21]). In *Drosophila melanogaster*, two p38 MAPK orthologs (p38a and p38b) participate in the host immune response to bacterial and fungal stimuli [19], while *Aedes* mosquitoes encode a single p38 MAPK that is involved in innate immune responses to bacterial pathogens [23–25]. The midgut/intestine is a central tissue for p38 MAPK regulation in both *D. melanogaster* [26–28] and in *C. elegans* [29, 30], where this pathway regulates gut homeostasis and immunity. The *Anopheles gambiae* [31] and *Anopheles stephensi* genomes each encode a single p38 MAPK, described herein, with associated pathway signaling proteins [31]. Based on collective observations from other species and the conservation of this pathway, we hypothesized that p38 MAPK signaling contributes to regulation of the response of *Anopheles* mosquitoes to midgut development of *Plasmodium* parasites [32].

In addition to regulating the innate immune response, p38 MAPK signaling also plays an important role in the coordination of cellular stress responses [33, 34]. For example, members of the MAPK family, including p38 MAPK, can be activated by reactive oxygen species (ROS) [31, 35, 36]. While high levels of ROS can be detrimental to the host and invading organisms, moderate levels of ROS can act as signaling mediators in a number of biological processes including both innate and adaptive immunity [37–39]. In *D. melanogaster,* ROS-activated p38 MAPK induces the expression of the antioxidant manganese superoxide dismutase (SOD2) [38], while in mammals activation of p38 MAPK by ROS increases expression of the antioxidants catalase and SOD [40]. In these organisms, the production of antioxidants acts to attenuate the molecular damage and cell death that high levels of ROS can cause and ultimately feeds back to inhibit p38 MAPK activity. However, in mosquitoes, high levels of ROS are not only detrimental to the host but also to invading organisms such as malaria parasites [8, 41, 42], indicating that a p38 MAPK-induced decrease in ROS could be beneficial to both host survival and parasite development. In addition, a number of studies have shown that ROS and NO can regulate activity of inflammatory signaling pathways including those that are dependent on nuclear factor (NF)-κB [43, 44], suggesting that p38 MAPK signaling could also alter ROS-dependent induction of NF-κB-dependent anti-malarial immune genes. Although we have previously shown that p38 MAPK can be activated by ROS in *Anopheles* midgut [31, 35], the downstream effects of this activation on mosquito biology remain unclear.

Activation of p38 MAPK has also been associated with increased protein translation via enhanced phosphorylation of MAPK-activated kinase 2 (MK2), which can increase mRNA stability and translation. In particular, MK2 abrogates mRNA instability caused by the presence of AU-rich elements in the 3'-untranslated region of transcripts [45–48]. In addition, p38 MAPK activation increases peroxisome proliferator-activated receptor gamma coactivator-1α (PGC-1α) phosphorylation and PGC-1β expression [48, 49]. PGC-1α and PGC-1β belong to a family of coactivator proteins that are highly responsive to environmental cues, such as temperature and nutritional status, and that regulate the maintenance of glucose, lipid, and energy (reviewed in [49]). In mammals, PGC-1α and PGC-1β regulate mitochondrial biogenesis and oxidative metabolism in a tissue- and trigger-specific manner through coactivation of the peroxisome proliferator-activated receptors [50, 51], estrogen-related receptors [52], and nuclear respiratory factors [53]. In addition to these functions, PGC-1α and PGC-1β regulate important aspects of metabolism and immunity. For example, PGC-1α, under starvation, exercise or adaptive thermogenesis, is known to drive expression of the gluconeogenetic enzymes phosphoenolpyruvate carboxykinase (PEPCK) and glucose-6-phosphatase (G6Pase), while PGC-1β enhances fatty acid oxidation and adrenergic-independent mitochondrial biogenesis, principally through the activation of pyruvate kinase (PK) and the hexokinase glucokinase [54–57]. In addition, PGC-1β has been recognized as a critical regulator of mammalian immune function [58]. In particular, interferon (IFN)-γ-induced activation of macrophage mitochondrial ROS for host defense is regulated via activation of PGC-1β by the IFN-γ-signal transducer and activator of transcription (STAT)-1 signaling cascade [59].

In *D. melanogaster*, the single PGC-1 ortholog Spargel controls mitochondrial biogenesis and is a downstream effector of the insulin-target of rapamycin (TOR) nutrient sensing signaling cascade, suggesting that PGC-1 control of energy homeostasis and nutritional status are conserved from insects to mammals [60–62]. Our previous studies have implicated coordinated control of insulin/insulin-like growth factor signaling (IIS)-dependent mitochondrial biogenesis with inflammatory ROS and NO synthesis and resistance to *P. falciparum* infection in *A. stephensi* [63]. The full spectrum of regulatory pathways, however, in the fly – and in anopheline mosquitoes as well – that integrate mitochondrial function and immunity is unknown. Collectively, our work and published observations suggested that a subset of these functions could be integrated by p38 MAPK signaling during the mosquito host response to infection. In support of this hypothesis, we show here that *P. falciparum* infection of *A. stephensi* enhances p38 MAPK activation in the midgut within 30 min of blood feeding. Pharmacological inhibition of *A. stephensi* p38 (*As*P38) MAPK signaling pathway yielded a significant decrease in *P. falciparum* development in the mosquito midgut. This was most likely due to increased ROS and enhanced immune gene expression observed within the first 24 h following inhibition of *As*P38 MAPK signaling. Increased ROS levels can directly kill parasites and signal anti-malarial immune gene expression. Concurrently, *As*P38 MAPK inhibition significantly reduced transcript levels of PGC-1 as well as expression of mitochondrial proteins related to ATP production, protein translation, and antioxidants, including mitochondrial SOD (SOD2). Functionally, inhibition of *As*P38 MAPK enhanced mosquito sensitivity to paraquat-induced oxidative stress and precipitated a shift to glycolytic metabolism even in the absence of parasite infection. Collectively these data suggest that *As*P38 MAPK-dependent repression of immunity to *P. falciparum* is associated with enhanced mitochondrial biogenesis, oxidative phosphorylation (OXPHOS), antioxidant biosynthesis, and protein translation, which may help the mosquito to survive parasite infection.

## Methods

### Identification of *A. stephensi* p38 MAPK

*Anopheles gambiae* p38 MAPK amino acid sequence [31] was used as a query to identify the homologous full-length coding sequence of the *As*P38 MAPK sequence in the February 2014 *A. stephensi* Indian strain assembly by Basic Local Alignment Search Tool (BLAST) [64]. The region with the best match and 1 kilobase of flanking sequence were retrieved. Encoded domain sequences were confirmed by manual protein sequence alignments. Putative translational start site of the assembled *As*P38 MAPK encoding gene was predicted using the ExPASy translate tool (web.expasy.org/translate/) and adherence to Kozak consensus [65].

RNA was isolated from 30 midguts of non-blood fed, female *A. stephensi* mosquitoes using TriZOL reagent (Invitrogen) for RNA extraction. Contaminating DNA was removed from the RNA samples using Turbo DNA-free (Ambion) and cDNA was synthesized using the SuperScript®III First-Strand Synthesis System (Invitrogen). Sample cDNAs were used to perform PCR using high fidelity AccuPrime Taq DNA Polymerase (Invitrogen). Primers were designed based on *As*P38 MAPK sequence using Primer Express software (Applied Biosystems). The following *As*P38 MAPK specific primers were used: *As*P38 MAPK forward 5’-GTGTGTCCATCTGTTCCGTA-3’ and reverse 5’-TTACGCCTGCGGTTC- 3’ for both cloning and sequencing.

### Cell culture and luciferase reporter assays

ASE cells were maintained as previously described [35]. For *As*P38 MAPK phosphorylation studies, ASE cells were either treated with 1 μg/ml LPS alone or pretreated with 10 μM BIRB796, 10 μM SB203580, or an equivalent volume of DMSO as a control for 2 h. Luciferase reporter assays were performed using the *Defensin1*, *Cecropin1*, and *Gambicin* promoter-reporter plasmids as previously described [35]. At 24 h post-transfection, cells were treated with 100 μg/ml LPS (Sigma-Aldrich) or 2 μg/ml peptidoglycan (PGN, *Escherichia coli* K12) with 0.1-10 μM BIRB796 or an equivalent volume of DMSO as a control. Luciferase activity was measured 24 h post-LPS or PGN treatment with the Dual-Glo system (Promega).

### Immunoblotting

Protein extracts were prepared, separated, and transferred to membranes as previously described [66]. Membranes were blocked in 5 % nonfat dry milk (w/v) in Tris-buffered saline (pH 7.0) containing 0.1 % Triton-100 (TBS-T) for 1 h at room temperature. Membranes were incubated at 4 °C overnight with the following: 1:2,500 rabbit anti-phospho-p38 MAPK antibody (Cayman Chemical), 1:2,000 rabbit anti-phospho-MK2 antibody (Abgent), or with 1:10,000 rabbit anti-GAPDH antibody (Abcam) in 5 % non-fat dry milk in TBS-T. Anti-phospho-p38 MAPK antibody and anti-phospho-MK2 antibody were raised against mammalian proteins; peptide sequences used to produce these monoclonal antibodies have greater than 90 % identity to their counterparts in *A. stephensi*. Membranes were washed three times for 5 min each in 1X TBS-T and incubated with a 1:20,000 dilution of HRP-conjugated goat anti-rabbit (Fab’)2 fragment (Cell Signaling Technology) at 4 °C overnight. To reveal antibody-bound proteins, membranes were incubated with SuperSignal West Dura chemiluminescent reagent for 5 min and visualized using the Kodak Image Station 4000MM Pro and Carestream Molecular Imaging software (Carestream Health). Levels of phospho-proteins in each treatment were first normalized to total protein levels as determined by GAPDH and then to the appropriate control group.

### Mosquito rearing and experimental treatments

*A. stephensi* Liston (Indian strain) were reared and maintained at 27 °C and 80 % humidity. All mosquito rearing and feeding protocols were approved and in accordance with regulatory guidelines and standards set by the Institutional Animal Care and Use Committee of the University of California, Davis. For experimental treatments, laboratory reared 3-5 day old female mosquitoes were kept on water overnight and then allowed to feed for 30 min on reconstituted human blood meals provided through a Hemotek Insect Feeding System (Discovery Workshops).

### *P. falciparum* culture and mosquito infection

For mosquito infection, cultures of *P. falciparum* strain NF54 MCB (obtained from Sanaria, www.sanaria.com) were grown as previously described [66]. Mosquitoes were allowed to feed on day 15 mature gametocyte cultures diluted with human RBCs and heat-inactivated human serum with or without the p38 MAPK inhibitors 0.1-10 μM SB203580 or 10 μM BIRB796. All treatments were added to the diluted *P. falciparum* culture immediately prior to blood feeding. Protocols involving the culture and handling of *P. falciparum* for mosquito feeding were approved and in accordance with regulatory guidelines and standards set by the Biological Safety Administrative Advisory Committee of the University of California, Davis. After 10 days, midguts from 50 mosquitoes with fully developed eggs (to confirm complete engorgement) from each group were dissected in PBS and stained with 0.1 % mercurochrome for direct counting of *P. falciparum* oocysts. Means of oocysts per midgut in each treatment group were calculated from all dissected mosquitoes, excluding zeros for mosquitoes that contained no oocysts.

### *P. falciparum* growth assays

Aliquots of *P. falciparum* NF54 culture were synchronized 48 h prior to the assay as described [67] and then plated in 96 well flat bottom plates in complete RPMI 1640 with HEPES, hypoxanthine and 10 % heat inactivated human serum. Parasites were treated with 1 or 10 μM SB203580 or BIRB796 or with an equivalent volume of DMSO diluent for 48 h in a candle jar in a 37 °C incubator. Infected RBCs were quantified as previously described [66].

### Quantitative real-time PCR of mRNA transcripts

For immune gene expression assays 3-5 day old *A. stephensi* were fed a blood meal containing freeze-thawed *P. falciparum* products (FTPP, preparation described in [68]) supplemented with 10 μM BIRB796 or an identical FTPP blood meal supplemented with an equivalent volume of DMSO diluent as a control. A total of 30 midguts at each time point were dissected and homogenized in TriZOL reagent (Invitrogen) for RNA extraction and contaminating DNA was removed from the RNA samples using Turbo DNA-free (Ambion). cDNA was synthesized from RNA samples using the SuperScript®III First-Strand Synthesis System (Invitrogen). Relative expression levels for *LRIM1*, *LRRD7*, *NOS*, *TEP1*, *DEF1* and *APL1* [68] were analysed with Maxima SYBR green/ROX qPCR Master Mix (Fermentas ThermoScientific) on an ABI 7300 Sequence Detection System (Applied Biosystems). Expression levels were calculated using the 2^−ΔΔCt^ method relative to the ribosomal protein s7 gene [9]. Data from biological replicates with separate cohorts of mosquito were used for statistical analysis.

### Preparation of *A. stephensi* for LC-MS/MS

Midguts from a total of 100 3-5 day old female *A. stephensi* fed with *P. falciparum*-infected blood and 10 μM BIRB796 or an identical infected blood meal supplemented with an equivalent volume of DMSO (diluent) as a control were collected 24 h post-blood feeding. Midgut samples were then lysed (50 mM Tris pH 6.8, 2 % SDS, 5 % Glycerol, 1 mM DTT, 3X protease and phosphatase inhibitor cocktail (Thermo Scientific) on ice for 50 min and centrifuged at 14,000 g/4 °C for 15 min. Protein concentrations in the supernatant were determined by BCA protein assay kit (Thermo Scientific) and 60 μg of total protein from each sample was electrophoretically separated by SDS-PAGE electrophoresis, and cut into 10 pieces. The following processes refer to the method in [63]. Specifically, when Scaffold (version Scaffold 3.5.1, Proteome Software Inc.) was used to validate MS/MS based peptide and protein identifications, peptide identifications were accepted if they could be established at greater than 95.0 % probability as specified by the Peptide Prophet algorithm [69]. Protein identifications were accepted if they could be established at greater than 99.0 % probability and contained at least 2 identified peptides. Protein probabilities were assigned by the Protein Prophet algorithm [70]. Proteins that contained similar peptides and could not be differentiated based on MS/MS analysis alone were grouped to satisfy the principles of parsimony. Using the parameters above, the False Discovery Rate (FDR) was calculated to be 0.3 % on the protein level and 0 % on the peptide level for the *A. gambiae* search set and 0.3 % on the protein level and 0 % on the peptide level for the *A. stephensi* search set [71]. Differential protein expression between BIRB796 and DMSO treatments were calculated using the Fisher’s exact t-test of the Scaffold program based on the total number of assigned spectra per protein.

### ROS assays

For detection of mitochondrial superoxide, groups of 50 (3-5 day old) female *A. stephensi* were held on water overnight, then allowed to feed for 30 min on an ATP-saline solution (150 mM NaCl, 10 mM NaHCO_3_ and 1 mM ATP) supplemented with equivalent volumes of 10 μM BIRB796 or DMSO as a control. Non-fed mosquitoes were removed at the end of 30 min. At 6 h post feeding, two pools of 10 midguts for each treatment were dissected and incubated in 150 μl of 5 μM MitoSOX Red (Molecular Probes-Life Technologies) in wells of a 96-well plate. The plate was incubated in the dark at room temperature for 30 min and then read at excitation 485 nm and emission 560 nm (Hidex Chameleon, LabLogic). Non-fed mosquito midguts were used to define baseline superoxide levels. Confocal images were prepared from additional midguts from the same feeding groups. For microscopy, midguts were mounted in Prolong Gold with DAPI (Molecular Probes), imaged using an Olympus FV1000 confocal microscope (Olympus) and processed using ImageJ [72] and Adobe Photoshop. Experiments were replicated four times with separate cohorts of mosquitoes.

For detection of midgut peroxides, 50 (3-5 day old) female *A. stephensi* were held on water overnight, and then allowed to feed for 30 min on an ATP-saline solution containing 2 mM paraquat (Sigma-Aldrich) supplemented with equivalent volumes of 10 μM BIRB796 or DMSO as a control. Pools of 5 dissected midguts in 50 μL PBS (two per treatment) were homogenized by brief sonication and centrifuged to pellet debris. Peroxide levels were estimated in 20 μL of sample supernatant in duplicate using the Pierce Quantitative Peroxide assay kit per manufacturer’s instructions. Samples were incubated for 20 min at room temperature prior to spectrophotometer reading. Peroxide assay experiments were replicated three times with separate cohorts of mosquitoes.

### Oxidative stress survivorship assays

100 female 3-5 day old *A. stephensi* were kept on water for 48 h, and then fed for 30 min on a saline/ATP solution supplemented with 1 mM paraquat (Sigma-Aldrich) and 10 μM BIRB796 or an equivalent volume of DMSO as a control. Dead mosquitoes were removed and counted from 8-72 h after feeding. The experiment was replicated three times with separate cohorts of mosquitoes.

### Pyruvate and lactate quantification

For quantification of pyruvate and lactate, 300 midguts/treatment were resuspended in 300 μl of ice-cold 5 % PCA and homogenized on ice with 4 × 30 s strokes with a hand homogenizer. The resulting suspension was centrifuged at 10,300 g for 5 min at 4 °C. The supernatants were collected and neutralized with 65 μl of 1 M K_2_CO_3_ visualized with 2.5 μl of 0.05 % methyl orange [73]. The neutralized extracts were then used for the quantification of pyruvate levels as described elsewhere [73], with minor modifications [74]. The assay was performed at 37 °C with 50 μl of neutralized extract and 200 μl of a reaction mixture consisting of 0.2 mM NADH in 0.2 M K_2_HPO_4_ (pH 8.0). The baseline was recorded at 340 nm for 3 min and then again for 10 min upon addition of 0.5 U/ml LDH. Levels of pyruvate were calculated using the NADH molar extinction coefficient (ε_340_ = 6,220 M^−1^ cm^−1^) and expressed as pmol/midgut.

Lactate levels were evaluated as described in [74] in the same neutralized supernatant used for pyruvate measurements. Final reaction volume was 200 μl and the assay was run at 37 °C. A total of 50 μl of midgut extract were added to 100 μl of glycine hydrazine buffer (prepared exactly as described in [73] and 1 mM NAD^+^. The baseline was recorded at 340 nm for 3 min, followed by addition of 2.5 U/ml LDH. Changes in absorbance were recorded for 10 min and lactate levels were calculated using the NADH molar extinction coefficient and expressed as pmol/midgut. Both pyruvate and lactate measurements were carried out in triplicate using a Tecan Infinite M200 microplate reader. Calibration curves carried out with sodium pyruvate and sodium lactate (0-2 μM) showed a detection limit of 0.6 and 0.3 μM, respectively, with a coefficient of variation < 3 %.

### Identification and characterization of *A. stephensi PGC-1*

BLAST analysis of the *A. gambiae* genome with the human PGC-1α gene sequence revealed AGAP004361 as the highest similarity sequence, sharing the characterized RNA recognition motif (RRM). *A. stephensi* ASTEI08687 is the ortholog of AGAP004361 as well as the ortholog of *D. melanogaster* Spargel/PGC-1 [60]. The homology between *A. stephensi* ASTEI08687 and Spargel is also greater than that between Spargel and human PGC-1α. Primers for *A. stephensi* ASTE002911 were designed using Primer Express software (Applied Biosystems): forward 5’-GGTGGTTCAAAGGCAAGTGT-3’ and reverse 5’-GCTTGTCTGGCTTGGCTATC-3’.

Midguts from *A. stephensi* fed with *P. falciparum*-infected blood supplemented with 10 μM BIRB796 or an equivalent volume of DMSO as a control were collected 6 and 24 h post-blood feeding. Midgut RNA was extracted and cDNA was synthesized as described above. Relative expression levels for *As*PGC-1 were analysed with Maxima SYBR green/ROX qPCR Master Mix (Fermentas ThermoScientific) on an ABI 7300 Sequence Detection System (Applied Biosystems). Expression levels were calculated using the 2^−ΔΔCt^ method relative to the ribosomal protein s7 gene [9]. Data from biological replicates with unique and separate groups of mosquito were used for statistical analysis.

### Statistical analyses

For parasite infection studies, data from three independent experiments with separate cohorts of mosquitoes were analysed for the main effects of experiment and treatment. Infection levels in controls from these replicates were not significantly different from one another (ANOVA), so these data were combined for statistical analysis. Differences in prevalence of infection (the presence of at least one oocyst in a dissected mosquito) between treatment conditions were determined by Fisher's exact test. For survival studies, the data were analyzed in GraphPad using Log-rank (Mantel-Cox) and Gehan-Breslow-Wilcoxon analysis. For all other data sets, significance was determined by Student’s t-test (alpha = 0.05) or with one-way ANOVA followed by Bonferroni’s post-test for multiple comparisons.

## Results

### Identification and characterization of *A. stephensi* p38 MAPK

**Fig. 1:**
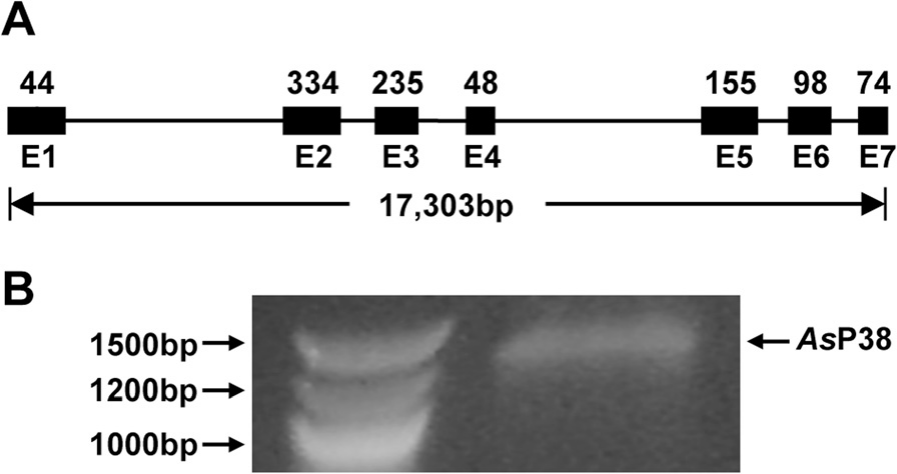
Gene structure and expression of *A. stephensi* p38 MAPK. (**a**) The *A. stephensi* p38 (*As*P38) MAPK gene includes 7 exons (black boxes; length in base pairs (bp) indicated), 6 introns, and spans 17,303 bp. (**b**) Total RNA was isolated and converted to cDNA from 30 dissected midguts from non-blood fed female *A. stephensi. As*P38 MAPK-specific primers were used to amplify cDNA by conventional PCR; molecular standards (bp) are shown on left

The *A. gambiae* p38 MAPK amino acid sequence [31] was used as a query to identify and annotate the full-length coding sequence of *As*P38 MAPK (ASTEI06041). As in *A. gambiae,* the *As*P38 MAPK genomic sequence spans seven exons and six introns (Fig. 1a), suggesting the existence of an ancestral p38 MAPK that predates the divergence of *Anopheles* spp. Full-length *As*P38 MAPK mRNA is expressed in the midgut epithelium of non-blood fed female *A. stephensi* (Fig. 1b). Across the length of encoded sequence, *As*P38 MAPK shares 74 % identity with human and mouse p38 MAPKs (not shown), allowing for the use of tools developed for mammalian p38 MAPKs (e.g., monoclonal antibodies, small molecule inhibitors) in our studies.

**Fig. 2:**
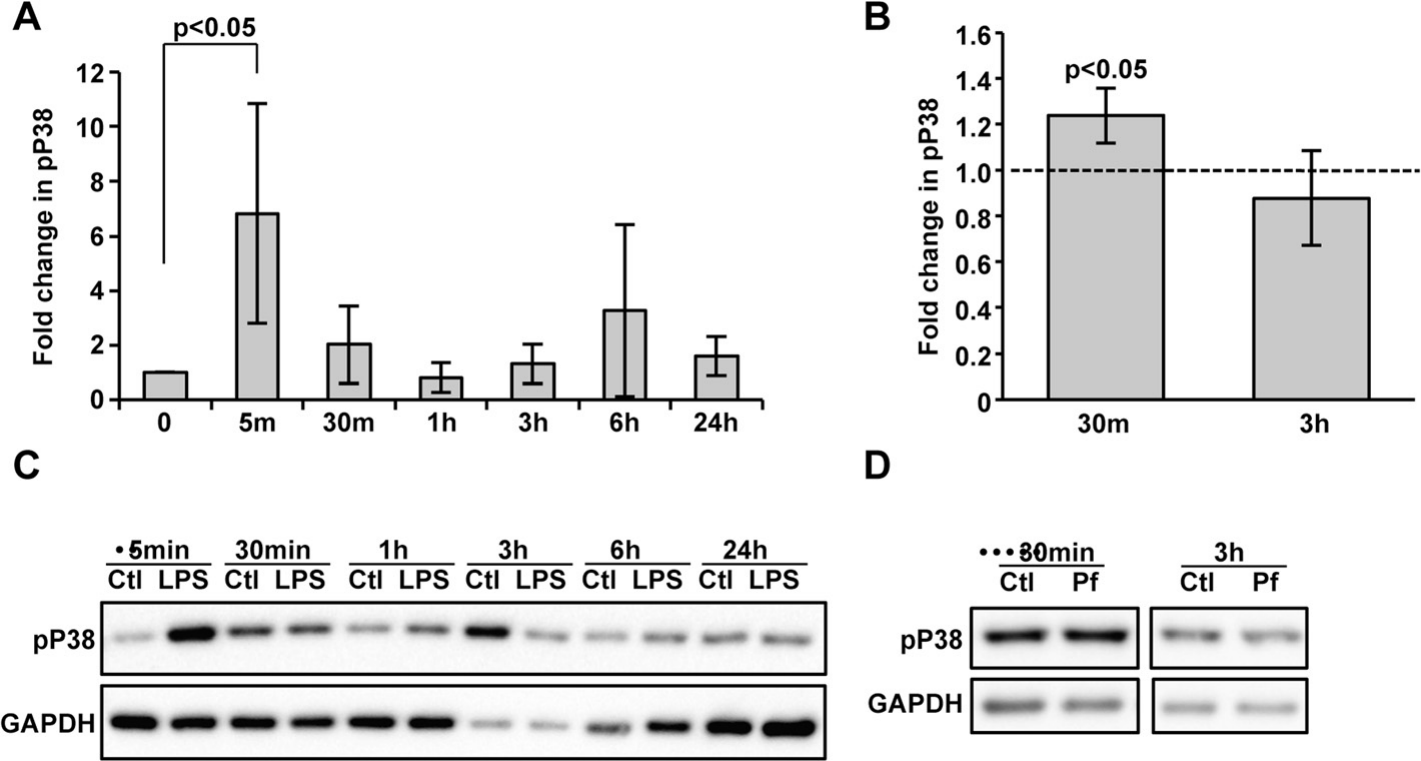
Phosphorylation of *As*P38 MAPK increased in response to LPS *in vitro* and to *P. falciparum* infection *in vivo*. (**a**, **c**) Cell lysates from ASE cells treated with 1 μg/ml LPS were collected at the indicated time points post-treatment and levels of phosphorylated p38 MAPK (pP38) were determined by western blotting. Graph in (**a**) represents average fold change ± SEMs of pP38 protein levels normalized to untreated controls, n = 3-4. GAPDH provided an assessment of protein loading. Pairwise comparisons of treatments versus controls at each time point were analysed by Student’s t-test, significant p-values are shown. (**c**) is a representative western blot. (**b**, **d**) Midgut cell lysates from 3-5 day old female *A. stephensi* mosquitoes fed uninfected or *P. falciparum*-infected blood were collected at 30 min and 3 h post feeding and levels of pP38 were determined by western blotting. Graph in (**b**) represents average fold change ± SEMs of pP38 protein levels normalized to uninfected blood fed controls, *n* = 3. Dotted line represents pP38 levels in uninfected blood fed controls. Pairwise comparisons of treatments versus controls at each time point were analysed by Student’s t-test, significant p-values relative to control are shown. (**d**) is a representative western blot

To determine whether *As*P38 MAPK phosphorylation and, hence, activation occurs in mosquito cells in response to stimulus with pathogen-associated molecular patterns (PAMPs), we used LPS as a prototypical bacterial PAMP and *Pf*Ps as a malaria parasite-specific stimulus. LPS can activate p38 MAPK signaling in the mosquito *Aedes aegypti* [23] and in *D. melanogaster* [19, 20, 37], so we reasoned that LPS should increase *As*P38 MAPK phosphorylation. Accordingly, immortalized *A. stephensi* embryonic (ASE) cells were stimulated with 1 μg/ml LPS and *As*P38 MAPK phosphorylation levels were measured over the course of 24 h. LPS stimulation resulted in a significant and rapid increase in phosphorylated *As*P38 MAPK that returned to levels not different from control by 30 min to 1 h after stimulation (Fig. 2a and 2b), confirming inducible p38 MAPK phosphorylation in *A. stephensi* that was analogous to that described in other organisms. In addition, ASE cells were stimulated with purified soluble *P. falciparum* products (*Pf*Ps, 36 parasite equivalents/cell) to confirm that parasite products alone could induce the *As*P38 MAPK pathway. *Pf*Ps was prepared as previously described [66] and tested negative for LPS contamination. We observed a significant increase in phosphorylated *As*P38 MAPK levels at 5 min post stimulation in response to *Pf*Ps that was reduced below control levels by 30 min (Additional file 1), confirming that parasite products, in the absence of LPS, can activate this pathway.

To determine whether *As*P38 MAPK was activated *in vivo* in response to malaria parasite infection*,* female *A. stephensi* were fed a blood meal containing *P. falciparum*-infected red blood cells (RBCs) and midgut *As*P38 phosphorylation levels were quantified at 30 min (immediately after the 30 min feeding) and 3 h post-blood feeding. The results revealed a significant increase in *As*P38 MAPK phosphorylation in mosquitoes fed *P. falciparum*-infected RBCs at 30 min post-feeding compared to mosquitoes fed RBCs alone that returned to baseline within 3 h (Figs. 2c and 2d), a pattern that was consistent with *Pf*Ps-induced *As*P38 phosphorylation *in vitro* (Additional file 1). Together, these results demonstrated that *As*P38 MAPK is activated in the mosquito midgut in response to *P. falciparum* infection.

### Inhibition of *As*P38 MAPK abolished phosphorylation of MAPK-activated kinase 2 (MK2) *in vitro* and *in vivo*

MAP kinase-activated protein kinase 2 (MK2) is a primary downstream kinase target of p38 MAPK and is commonly used to confirm p38 MAPK activity. Studies in mammals have confirmed that activated MK2 mediates p38 MAPK-dependent stabilization and efficient translation of short-lived mRNAs (reviewed in [75]). Highly homologous MK2 proteins are encoded in the *A. gambiae* (AGAP011890) and *A. stephensi* (ASTEI05089) genomes, suggesting that a conserved p38 MAPK-MK2 pathway exists in these species. To establish that MK2 is a conserved target of *As*P38 MAPK activation, we selected the commonly used p38 MAPK small molecule inhibitors SB203580 [76] and BIRB796 [77]. Unlike SB203580, which directly competes with ATP binding to block kinase activity, BIRB796 induces a conformational change in p38 MAPK making it incompatible with ATP-binding and abolishing its kinase activity [78]. The residues in the active site of p38 MAPK that are necessary for BIRB796 binding are 100 % conserved in *A. stephensi* [78], as is the ATP binding site that SB203580 interacts with (not shown) [79]. BIRB796 also blocks p38 MAPK phosphorylation and p38 MAPK nuclear localization that occurs in response to DNA damage [80]. We used SB203580 and BIRB796 side-by-side in a number of assays to confirm targeting of *As*P38 MAPK (and lack of an effect on *P. falciparum* growth, see below), but carried forward with BIRB796 in our remaining assays because of its minimal off-target effects [78] at the concentrations used in this study.

**Fig. 3:**
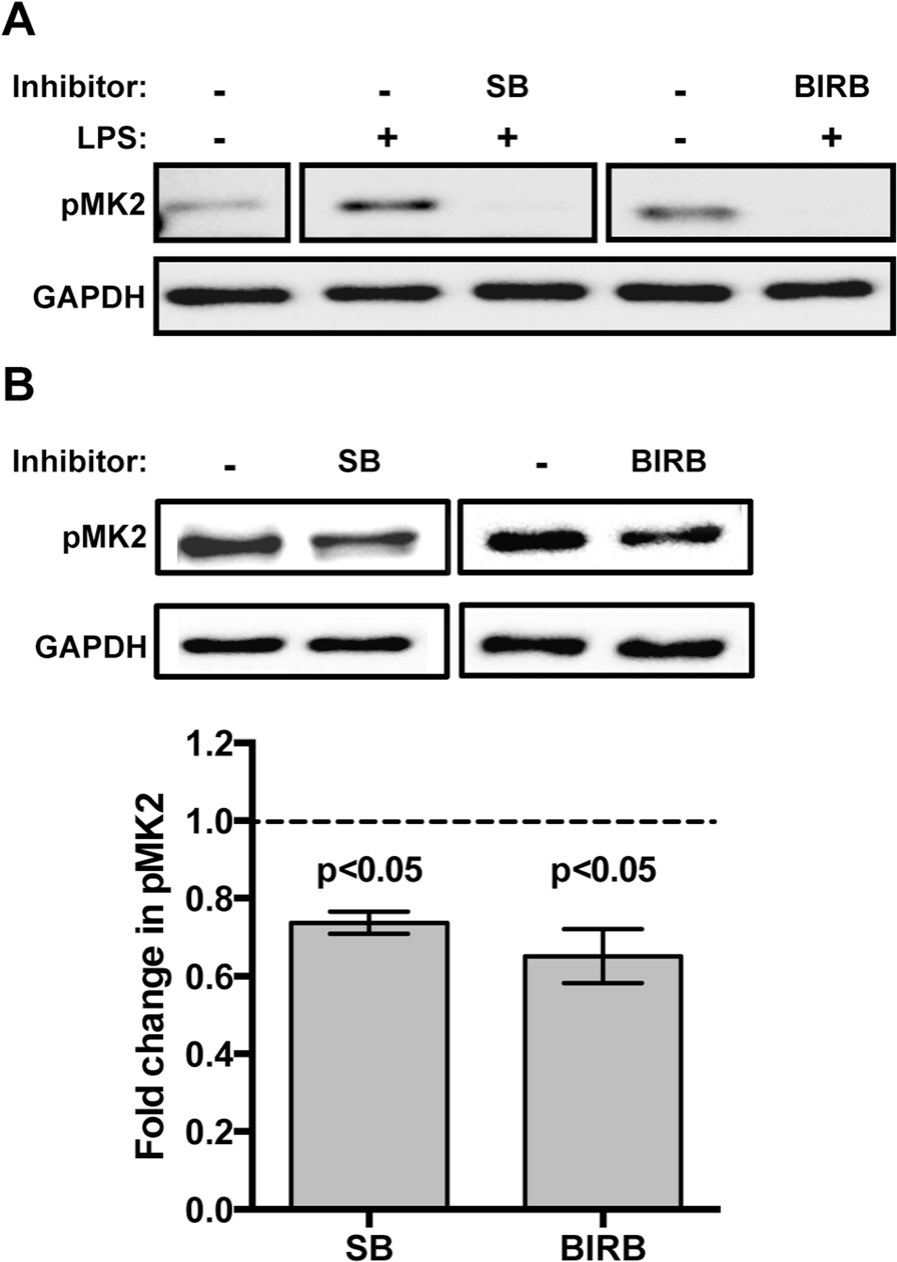
Inhibition of *As*P38 MAPK reduced phosphorylation of MAPK-activated kinase 2 (MK2) *in vitro* and *in vivo*. **a** Representative western blot of phosphorylated MK2 (pMK2) from ASE cells pretreated with 10 μM SB203580 (SB), 10 μM BIRB796 (BIRB), or an equivalent volume of DMSO as a control for 2 h and then stimulated with 1 μg/ml LPS for 15 min. GAPDH provided an assessment of protein loading. **b** Representative western blot of phosphorylated midgut MK2 (pMK2) from *A. stephensi* fed a *P. falciparum*-infected blood meal supplemented with 10 μM SB203580 (SB), 10 μM BIRB796 (BIRB), or an equivalent volume of DMSO; tissues were dissected at 2 h post-feeding for analysis. Graph represents fold change ± SEMs of pMK2 protein levels normalized to untreated controls, *n* = 3. Pairwise comparisons of treatments versus controls at each time point were analysed by Student’s t-test, significant p-values are shown

Treatment with either SB203580 or BIRB796 completely abrogated MK2 phosphorylation in ASE cells *in vitro* in response to 1 μg/ml LPS (Fig. 3a), establishing the efficacy of these small molecule inhibitors for use in *A. stephensi* and confirming that MK2 is a phosphorylation target of *As*P38 MAPK during PAMP activation. Based on the different modes of action of these inhibitors, these results also confirmed that the observed effects were not influenced by local concentrations of ATP that may have obscured the inhibition pattern of *As*P38 MAPK. Provision of SB203580 and BIRB796 in a *P. falciparum*-infected blood meal reproducibly reduced, but did not completely inhibit, MK2 phosphorylation in the *A. stephensi* midgut at 2 h after feeding (Fig. 3b).

### Inhibition of *As*P38 MAPK decreased *P. falciparum* development in *A. stephensi*

**Fig. 4:**
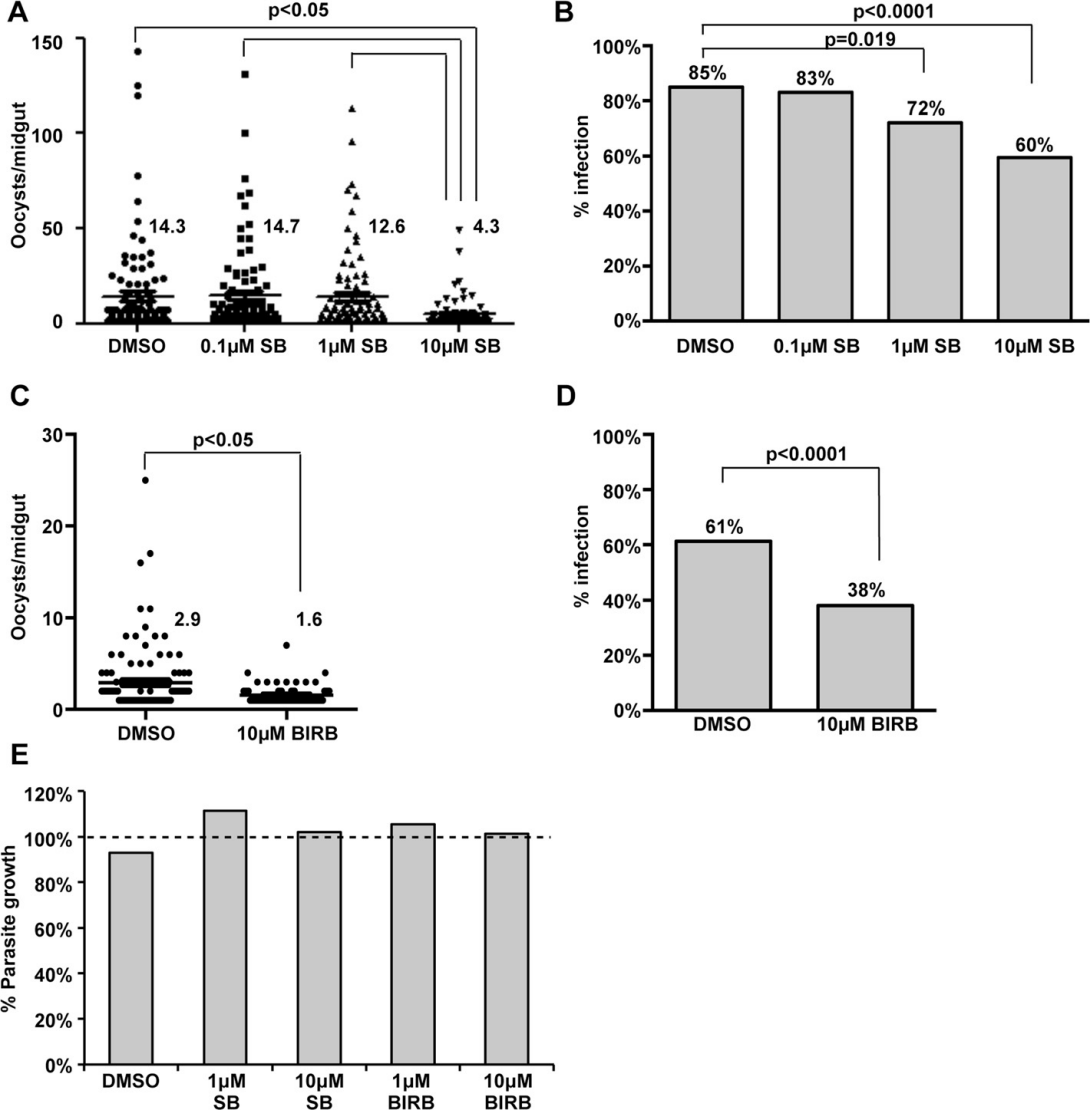
Inhibition of *As*P38 MAPK signaling reduced *P. falciparum* development in *A. stephensi*. Mosquitoes were provided with a *P. falciparum*-infected blood meal supplemented with (**a**, **b**) 0.1-10 μM SB203580 (SB) or (**c**, **d**) 10 μM BIRB796 (BIRB) or an equivalent volume of DMSO as a control. Midguts were dissected and oocysts were counted at 10 days following infection. The experiment was replicated three times with separate cohorts of mosquitoes and analysed by ANOVA to determine if the oocyst intensity in the controls differed amongst replicates. No difference was found, and the data were pooled across replicates and analysed by Kruskal-Wallis to test for overall significance and Dunn's post-test for pairwise comparisons of means. Significant p-values are shown for mean oocysts per infected midgut (zero values excluded). Prevalences of infection (mosquitoes with at least one *P. falciparum* oocyst) are shown as percentages of dissected mosquitoes. Fisher's exact test was used to test for significance. (**e**) *P. falciparum* cultures were incubated with 1 μM or 10 μM of SB203580 or BIRB796 or equivalent volume of DMSO as a control for 48 h. Graph represents average relative growth compared to untreated controls (dotted line) at 48 h, *n* = 3. Pairwise comparisons of treatments versus control were analysed by Student’s t-test. No significant differences among control and treatment groups were found

Having confirmed that *As*P38 MAPK is activated by *P. falciparum* infection, we sought to determine the impact of *As*P38 MAPK-dependent signaling on *P. falciparum* development in the mosquito. Female *A. stephensi* mosquitoes were provided identical infectious blood meals enriched with *P. falciparum* gametocytes that were supplemented with 0.1-10 μM SB203580, 10 μM BIRB796, or with an equivalent volume of DMSO as a diluent control and the number of oocysts per midgut were quantified at 10 days post-blood feeding. Two-way ANOVA for each inhibitor indicated no significant differences in control infection levels among replicates, thus data from the replicates were combined for analysis. Treatment with 10 μM SB203580 resulted in a 70 % decrease in infection intensity from 14.3 to 4.3 oocysts per midgut (Fig. 4a). Prevalence of infection (the presence of at least one oocyst in a dissected mosquito) decreased significantly from 85 % to 60 % following treatment with 10 μM SB203580 (Fig. 4b). Treatment of mosquitoes provided infectious blood meals supplemented with 10 μM BIRB796 also yielded significant reductions in infection intensity and prevalence (Fig. 4c and 4d). These data suggested that *As*P38 MAPK-dependent signaling alters mosquito biology in a manner that benefits malaria parasite development.

Although *P. falciparum* does not encode a prototypical p38 MAPK, the parasite genome does encode two MAPKs: Pfmap-1 which is orthologous to ERK7/8 and Pfmap-2 which is not directly orthologous to any mammalian MAPK subfamilies [81]. In addition, some p38 MAPK inhibitors have been shown to reduce the replication of asexual stage *P. falciparum* [82]. Therefore, we sought to confirm that the observed effects on oocyst development in *A. stephensi* were due to inhibition of mosquito *As*P38 MAPK signaling rather than inhibitor-induced alterations of the intrinsic growth of the parasite. To this end, we quantified DNA content over time as a measure of growth of synchronized asexual *P. falciparum* parasites treated with 1 and 10 μM SB203580 and BIRB796 *in vitro*. We observed no significant effects on parasite growth following treatment with either inhibitor (Fig. 4e). Although this growth assay cannot be performed efficiently on mosquito-stage parasites, we infer from these data that the infection patterns observed *in vivo* using p38 MAPK inhibitors are likely not due to direct negative effects on parasite development in the mosquito. However, given the limitations of our assay, we cannot rule out the possibility that these inhibitors could interfere with ookinete development, parasite migration through the epithelium, or the development of oocysts and sporozoites.

### Inhibition of *As*P38 MAPK altered antimicrobial peptide promoter activity in response to LPS or peptidoglycan (PGN) stimulation in immortalized *A. stephensi* cells

**Fig. 5:**
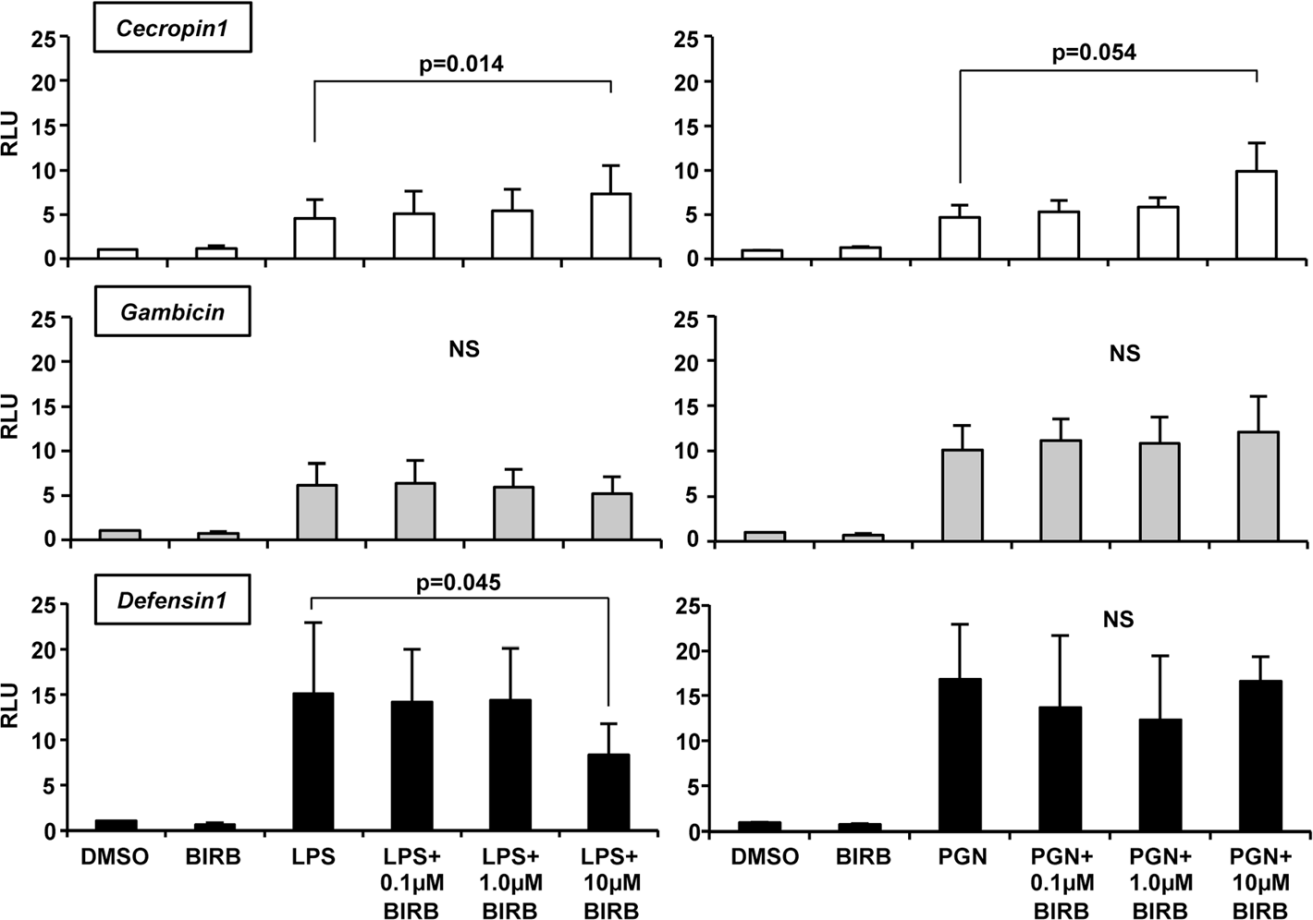
Inhibition of *As*P38 MAPK altered antimicrobial peptide promoter activity in response to LPS or PGN stimulation in immortalized *A. stephensi* cells. ASE cells were transfected with an antimicrobial peptide luciferase promoter-reporter plasmid construct and stimulated 24 h later with 100 μg/ml LPS or 2 μg/ml PGN with 0.1 -10 μM BIRB796 or an equivalent volume of DMSO as a control. Graphs represent means ± SEMs of luciferase activity (relative light units, RLU) normalized to untreated controls, *n* = 3-7. *Cecropin1* (top, white) or *Gambicin* (middle, gray), or *Defensin1* (bottom, black) promoter activities are indicated. Data were analysed by Student’s t-test and significant p-values are shown. NS = not significant

Because p38 MAPK signaling is critical to innate immune responses [83], we sought to determine the extent to which *As*P38 MAPK could regulate NF-κB-dependent promoter activity in mosquito cells *in vitro*. We utilized a luciferase-reporter assay to quantify the response of mosquito cells *in vitro* to the bacterial PAMPs LPS and peptidoglycan (PGN). ASE cells were stimulated with either LPS or PGN and promoter activities of the NF-κB-dependent antimicrobial peptide (AMP) genes *Defensin1*, *Cecropin1*, and *Gambicin* were measured in the presence of vehicle (DMSO) or following pre-treatment with 0.1-10 μM of BIRB796 in DMSO. All three promoter-reporters were induced by LPS and PGN as previously described [7, 66, 68] (Fig. 5). As expected, treatment with BIRB796 alone did not alter activity of the *Defensin1*, *Cecropin1*, or *Gambicin* promoters in ASE cells (Fig. 5). Further, although BIRB796 pre-treatment had no effect on *Gambicin* promoter activity (Fig. 5, middle panel), pre-treatment with 10 μM BIRB796 significantly increased LPS- and PGN-dependent *Cecropin1* promoter activity (Fig. 5, top panel) and significantly decreased LPS-dependent *Defensin1* promoter activity (Fig. 5, bottom panel), suggesting that the pattern of *As*P38 MAPK regulation of *A. stephensi* antimicrobial peptide expression *in vitro* are gene- and stimulus-specific.

### Inhibition of *As*P38 MAPK increased parasite-inducible expression of immune genes in the *A. stephensi* midgut

**Fig. 6:**
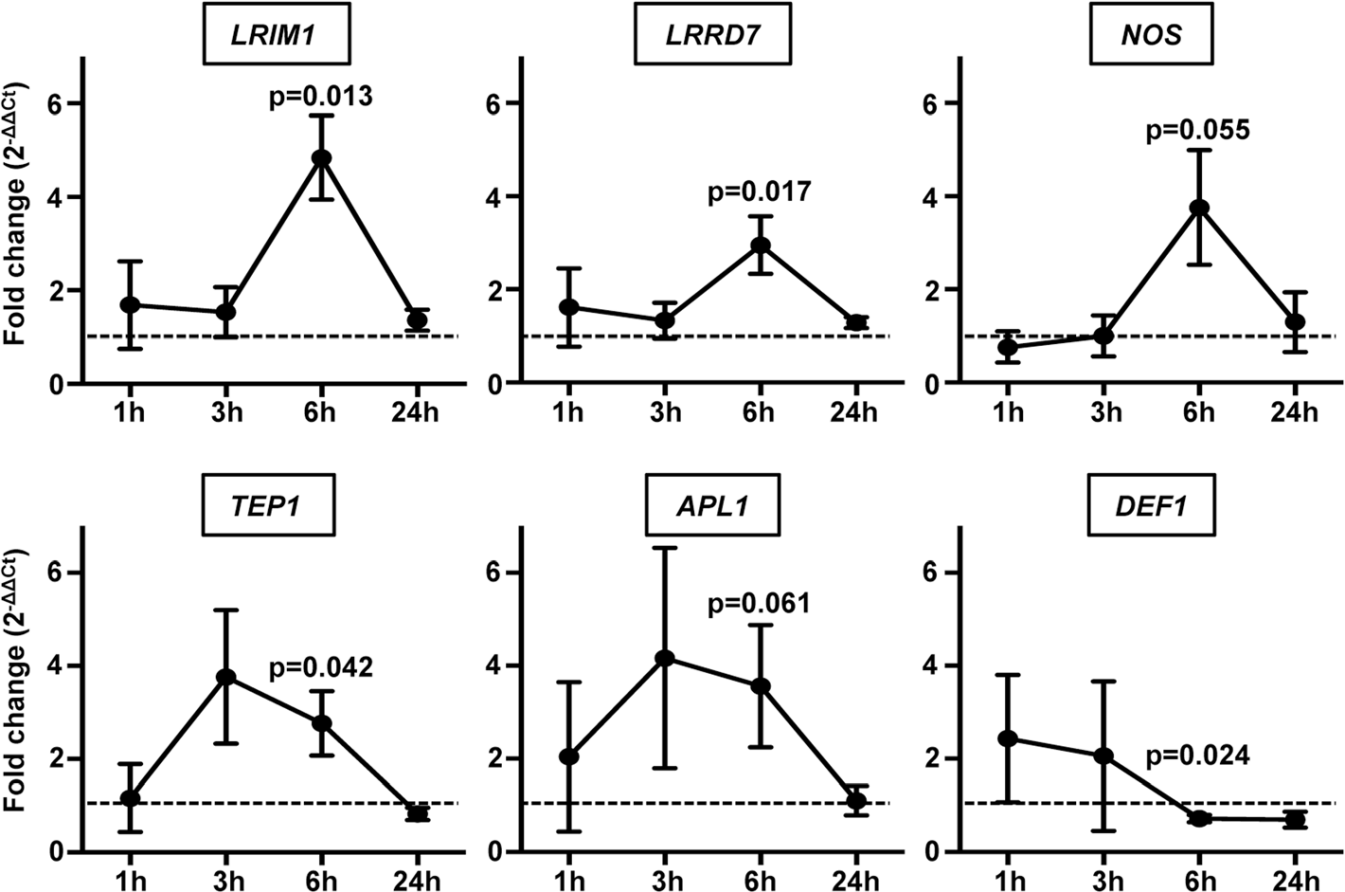
Inhibition of *As*P38 MAPK increased parasite-inducible expression of immune genes in the *A. stephensi* midgut. Graphs represent means ± SEMs of fold change in the expression of selected immune genes in the midgut of mosquitoes fed a blood meal with *P. falciparum* freeze/thaw parasite products (FTPP) supplemented with 10 μM BIRB796 or an identical FTPP meal supplemented with an equivalent volume of DMSO as a control. Pairwise comparisons of treatments and matched controls were analysed by Student’s t-test; significant and marginally significant p-values are shown, *n* = 3-5

Our *in vitro* data indicated that *As*P38 MAPK both positively and negatively regulated the expression of antimicrobial peptide genes, suggesting that activation of this pathway facilitates parasite development *in vivo* via changes in anti-parasite effector genes. To test this hypothesis, we used gene-specific primers to examine midgut transcript expression levels of *nitric oxide synthase* (*NOS*; ASTE008593*), LRIM1* (ASTE000814)*, TEP1* (ASTE010227)*, APL1* (ASTEI02571)*, LRRD7/LRIM17* (ASTE009590) and *Defensin1 (DEF1*; ASTE011281*),* following blood meals containing *P. falciparum* freeze/thaw parasite products (FTPP) in the presence or absence of 10 μM BIRB796 over the course of 24 h. Inhibition of *As*P38 MAPK activity induced the expression of almost all anti-malarial effector genes at 6 h (only *NOS* and *APL1* were marginally not significant), with a return to baseline expression levels by 24 h after feeding (Fig. 6)*.* In contrast, *DEF1* gene expression was decreased after *As*P38 MAPK inhibition, consistent with *in vitro* promoter activity following LPS stimulation (Fig. 5). These data suggested that *As*P38 MAPK-dependent signaling negatively regulates the activation of immune genes associated with parasite killing, providing a potential mechanistic explanation for the decrease in parasite development observed following inhibition of MAPK activity (Fig. 4).

### Inhibition of *As*P38 MAPK decreased mosquito antioxidant defenses

In addition to its role in the regulation of innate immunity, p38 MAPK signaling can also affect stress responses (reviewed in [84]). To understand these and the other potential roles of *As*P38 MAPK signaling on a more global level, differential quantitative liquid chromatography/tandem mass spectrometry (LC-MS/MS) was performed to identify proteins that were over- or underrepresented in *A. stephensi* fed *P. falciparum*-infected blood supplemented with 10 μM BIRB796 relative to age-matched, female *A. stephensi* fed an identical infected blood meal supplemented with an equivalent volume of DMSO as a control. At 24 h post-feeding, 13 proteins out of a total of 1,418 proteins were over-represented and 60 were underrepresented following inhibition of *As*P38 MAPK (Table 1). These results suggested that *As*P38 MAPK promotes protein translation and synthesis as indicated in the literature [45–48].

**Table 1:** Proteins over- and under-represented in the *A. stephensi* midgut following inhibition of *As*P38 MAPK signaling

Over-represented proteins following inhibition of *As*P38 MAPK				
Pathway or process	Accession number	Protein name	UniProt gene name	*P* value
Carbohydrate metabolism	ASTEI05125	maltase 2 (mal2)	MGAM	0.011
	ASTEI02309	maltase	MGAM	0.007
Cytoskeleton/development	ASTEI01099	thymosin	TMSNB	0.041
	ASTEI03296	dynamin	DNM	0.023
	ASTEI04270	gelsolin	GSN	0.036
	ASTEI05229	collagen alpha-1 chain	COLA1	0.008
	ASTEI08311	myosin	MYH	0.001
	ASTEI11230	coracle	CORA	0.001
Lysosomes	ASTEI08520	v-type proton ATPase subunit c	VATC	0.023
Mitochondria	ASTEI11442	NADH dehydrogenase 1 beta subcomplex subunit 10	NDUFB10	0.020
	ASTEI08224	60 kDa heat shock mitochondrial	HSP60	0.006
Membrane fusion/exocytosis	ASTEI07544	synaptosomal-associated protein 25	SNAP25	0.003
	ASTEI07916	Nipsnap	NIPSNAP1	0.008
Under-represented proteins following inhibition of *As*P38 MAPK				
Pathway or process	Accession number	Protein name	UniProt gene name	*P* value
Glycolysis	ASTEI06800	hexokinase type 2	HK2	0.023
	ASTEI05709	triosephosphate isomerase	TPI1	0.019
	ASTEI07469	pyruvate kinase	PKM	0.009
Pentose phosphate shunt	ASTEI07641	6-phosphogluconate dehydrogenase	PGD	0.027
Fatty acid biosynthesis/quinone reductase	ASTEI07966	3-ketoacyl-acyl carrier protein reductase	FABG	0.009
Mitochondrial ketone body metabolism	ASTEI05771	succinyl-ligase subunit	SUCLA2	0.034
Mitochondrial OXPHOS	ASTEI01282	succinate dehydrogenase iron-sulfur	SDHB	0.052
	ASTEI05686	NADH-ubiquinone oxidoreductase/75 kD	NDUFS1	0.029
	ASTEI10851	cytochrome *c* oxidase subunit vIIa	COX7A1	0.034
	ASTEI09954	ATPase subunit F	ATP5F	0.004
	ASTEI02184	3-hydroxybutyrate dehydrogenase	HIBADH	0.047
Mitochondrial Krebs cycle	ASTEI01282	succinate dehydrogenase iron-sulfur	SDHB	0.052
Mitochondrial protein import/processing	ASTEI04325	putative inner membrane protein	IMMT	0.012
	ASTEI06526	porin	VDAC	0.005
Mitochondrial dynamics	ASTEI08858	kinesin heavy chain	KIF5B	0.009
Mitochondrial antioxidant defenses	ASTEI07113	superoxide dismutase 2	SOD2	0.006
Ribosomes/translation	ASTEI06447	40s ribosomal protein s16	RPS16	0.053
	ASTEI09012	40s ribosomal protein s19a	RPS19A	0.015
	ASTEI08714	40s ribosomal protein s3	RPS3	0.017
	ASTEI09336	40s ribosomal protein s3a	RPS3A	0.021
	ASTEI09193	40s ribosomal protein sa	RPSA	0.006
	ASTEI01139	60s ribosomal protein L10	RPL10	0.026
	ASTEI04204	60s ribosomal protein L10a	RPL10A	0.015
	ASTEI00128	60s ribosomal protein L13	RPL13	0.028
	ASTEI09035	60s ribosomal protein L15	RPL15	0.001
	ASTEI01482	60s ribosomal protein L18	RPL18	0.053
	ASTEI07898	60s ribosomal protein L23	RPL23	0.022
	ASTEI08588	60s ribosomal protein L27a	RPL27A	0.002
	ASTEI05093	60s ribosomal protein L31	RPL31	0.020
	ASTEI00059	60s ribosomal protein L36	RPL36	0.038
	ASTEI00185	60s ribosomal protein L4	RPL4	0.007
	ASTEI10247	60s ribosomal protein L5	RPL5	0.001
	ASTEI01907	60s ribosomal protein L7	RPL7	0.010
	ASTEI063681	heterogeneous nuclear ribonucleoprotein 40	HNRNPH1	0.034
	ASTEI05590	polyadenylate-binding protein	PABP	0.048
	ASTEI08624	nascent polypeptide-associated complex subunit alpha	NACA	0.027
	ASTEI11090	signal recognition particle receptor, alpha homolog	SRPR	0.033
	ASTEI02606	eukaryotic translation initiation factor 3 subunit e	EIF3E	0.038
	ASTEI02686	eukaryotic translation initiation factor 3 subunit i	EIF3I	0.039
	ASTEI012841	RNA-binding protein 1	RBP1	0.005
	ASTEI06323	ribosomal protein l14	RPL14	0.000
	ASTEI06775	rRNA 2-o-methyltransferase fibrillarin	FBL	0.022
	ASTEI01829	cchc-type zinc finger protein	ZCRB1	0.034
	ASTEI10281	nipped-b protein	NIPBL	0.017
Antioxidant/detoxification	ASTEI10644	catalase	CAT	0.022
	ASTEI07113	superoxide dismutase 2	SOD2	0.006
	ASTEI03473	aldehyde dehydrogenase family 7 member a1 homolog	ALDH7A1	0.003
	ASTEI06895	cytochrome *b*_5_	CYB5	0.040
Proteolysis/lysosomes	ASTEI07459	membrane alanyl aminopeptidase	ANPEP	0.001
	ASTEI09440	hypodermin c		0.045
	ASTEI10033	chymotrypsin-2	CTRB1	0.008
	ASTEI07899	proteasome subunit alpha type-1	PSMA1	0.039
	ASTEI00715	endochitinase	CHIT1	0.045
	ASTEI10979	v-atpase subunit h	ATP6V1H	0.047
	ASTEI07458	aminopeptidase n	ANPEP	0.027
	ASTEI03577	acylsphingosine deacylase	ASAH1	0.012
Endocytosis	ASTEI09008	Ras-related protein Rab-18-b	RAB18	0.017
	ASTEI10979	v-atpase subunit h	ATP6V1H	0.047
	ASTEI03296	dynamin	DNM	0.023
	ASTEI01514	ocia domain-containing protein 1	OCIAD1	0.039
Iron storage	ASTEI00707	ferritin heavy chain	FTH1	0.031
Signal transduction	ASTEI02790	guanine nucleotide-binding protein subunit beta-1	GNB1	0.003
	ASTEI00708	protein ubash3a homolog	UBASH3A	0.016
	ASTEI05550	phosphatidylethanolamine-binding protein	PEBP1	0.005

**Fig. 7:**
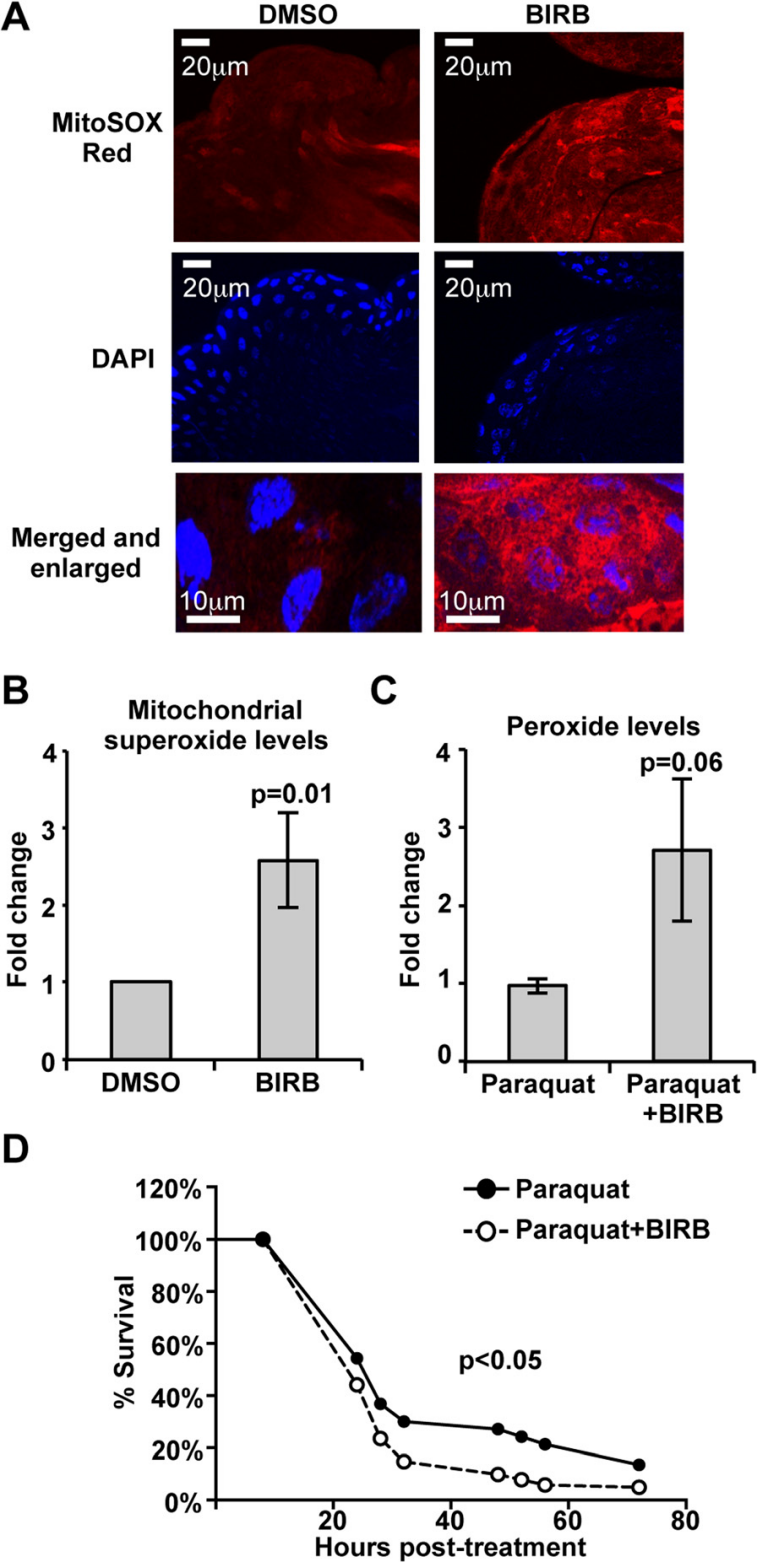
Inhibition of *As*P38 MAPK in *A. stephensi* increased midgut ROS levels and reduced mosquito survival. *A. stephensi* females were fed a saline solution containing either 10 μM BIRB796 or an equivalent volume of DMSO as a control and 10 midguts/group were collected 6 h post-feeding. **a** Representative images from confocal microscopy of MitoSOX Red stained midguts. Upper Panel: MitoSOX Red staining, scale bars = 20 μm; Middle Panel: DAPI staining scale bars = 20 μm; Lower Panel: Merged images, scale bars = 10 μm. **b** Graph of *A. stephensi* midgut mitochondrial superoxide levels as determined by Mitosox Red fluorescence staining. Data were analysed by Student’s t-test and are represented as means ± SEM, p-value is shown, *n* = 4. **c** Graph of midgut peroxide levels in *A. stephensi* fed with saline solution containing 2 mM paraquat with 10 μM BIRB796 or an equivalent volume of DMSO as a control at 6 h post-feeding. Data were analysed by Student’s t-test and represented as means ± SEM, p-value is shown, *n* = 3. **d** Representative survivorship curve from Table 2 of *A. stephensi* fed a saline solution with 1 mM paraquat supplemented with 10 μM BIRB796 or with an equivalent volume of DMSO as a control

Among the underrepresented proteins, catalase and SOD2 were significantly reduced by *As*P38 MAPK inhibition (Table 1). SOD2 is a mitochondrial manganese SOD that dismutates superoxide anion into hydrogen peroxide and diatomic oxygen; therefore, a reduction in SOD2 levels should result in increased midgut steady-state concentrations of mitochondrial superoxide. To test this hypothesis, we estimated superoxide concentrations in midgut mitochondria of female *A. stephensi* fed a saline solution containing either 10 μM BIRB796 or a saline solution with an equivalent volume of DMSO as a control. At 6 h post-feeding, mitochondrial superoxide levels in BIRB796-fed mosquitoes were significantly higher than controls (Fig. 7a and 7b). Unmitigated oxidative stress has also been associated with decreased short-term survivorship in p38 MAPK mutants of *D. melanogaster* [38]. Accordingly, we tested whether reduced antioxidant defenses (SOD2, catalase) could be associated with reduced mosquito survivorship in the context of oxidative stress via feeding of a saline meal with 2 mM paraquat (an inducer of superoxide anion production via redox cycling [85]) in DMSO or with 2 mM paraquat and 10 μM BIRB. As expected, midgut peroxide levels relative to controls were increased, albeit marginally significantly, by inhibition of *As*P38 MAPK (Fig. 7c). Inhibition of *As*P38 MAPK also resulted in significantly reduced survivorship to acute paraquat challenge compared to the controls (Fig. 7d, Table 2). Taken together, these results indicated that *As*P38 MAPK signaling likely enhances mosquito resistance to oxidative stress and that inhibition of this MAPK pathway significantly decreases ROS catabolism.

**Table 2:** Inhibition of *As*P38 MAPK under conditions of oxidative stress reduces survival of adult female *A. stephensi*

	Median survival time (hours)		Log-rank (Mantel-Cox)	Gehan-Breslow-Wilcoxon
Exp	Paraquat + DMSO	Paraquat + BIRB	p-values	p-values
1	28	24	0.004	0.026
2	28	24	0.015	0.007
3	32	24	0.025	0.052

### Increased mitochondrial ROS induced expression of *A. stephensi* immune genes

**Fig. 8:**
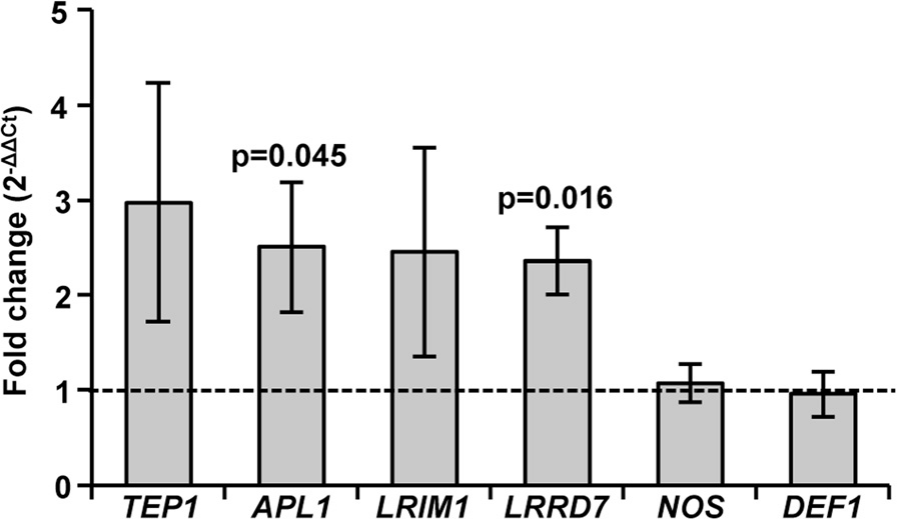
Mitochondrial ROS induced expression of *A. stephensi* immune genes. Graph represents means ± SEMs of fold change in the expression of selected immune genes in RNA samples prepared from 30 pooled midguts of mosquitoes fed a blood meal containing 1 μM rotenone relative to mosquitoes fed an unsupplemented blood meal (dotted line) at 6 h post-feeding. Data were analysed by Student’s t-test, significant p-values are shown, *n* = 3

In addition to the known ability of high levels of ROS to directly kill malaria parasites [86, 87], moderate levels of ROS can signal in the midgut to activate the mosquito immune response [35, 41, 88]. Therefore, we reasoned that the increase in anti-malarial effector gene expression observed following inhibition of *As*P38 MAPK (Fig. 6) could be due, in part, to increased mitochondrial ROS (Fig. 7a and b). To assess the contribution of mitochondrial ROS to the activation of *A. stephensi* immune genes, mosquitoes were fed a blood meal containing 1 μM rotenone, a concentration that can induce mitochondrial superoxide in the *A. stephensi* midgut to approximately 2.5-fold that of control levels [89], consistent with BIRB-induced superoxide levels (Fig. 7b). At 3 h post-feeding, midguts were collected for analysis of anti-malarial immune gene expression by qRT-PCR. Rotenone treatment alone significantly increased the expression of *APL1* and *LRRD*7 with trends toward increased *TEP1* and *LRIM1* expression relative to controls (Fig. 8). However, no increased expression was detected for *NOS* or *DEF1* with rotenone treatment (Fig. 8), suggesting that mitochondrial ROS contributes to, but does not fully recapitulate, BIRB796-induced immune gene expression in the *A. stephensi* midgut.

### Inhibition of *As*P38 MAPK decreased *AsPGC-1* gene expression and mitochondrial protein synthesis in *A. stephensi*

**Fig. 9:**
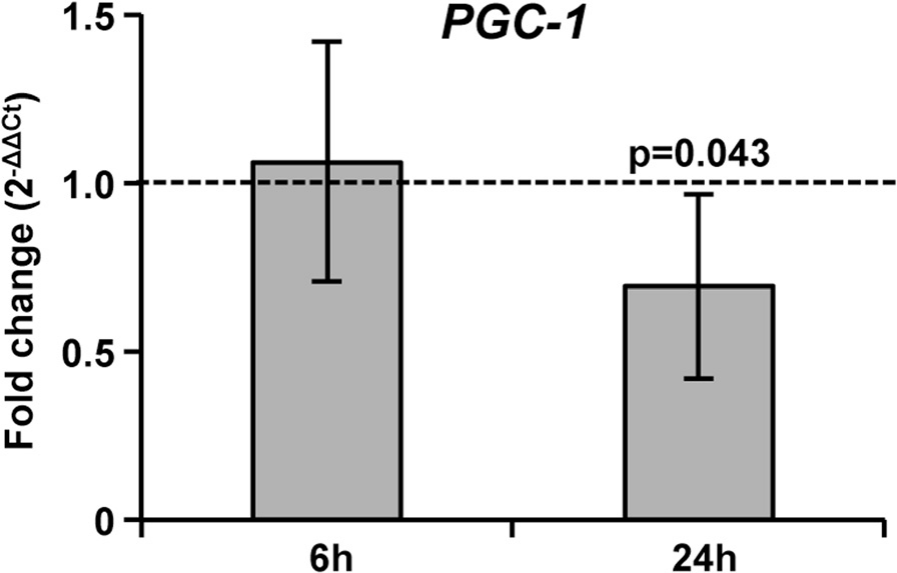
Inhibition of *As*P38 MAPK decreased As*PGC-1* gene expression in the *A. stephensi* midgut. Mosquitoes were fed with *P. falciparum*-infected RBCs supplemented with 10 μM BIRB796 (BIRB) or an equivalent volume of DMSO as a control (dotted line). Graph represents means ± SEMs of fold change in the expression of As*PGC-1* in RNA prepared from 30 pooled mosquito midguts, *n* = 5. Data were analysed by Student’s t-test; significant p-values are shown

Inhibition of *As*P38 MAPK was associated with significantly reduced levels of midgut mitochondrial proteins, including NADH-ubiquinone oxidoreductase (Complex I), succinate dehydrogenase (Complex II), cytochrome *c* oxidase subunit VIIa (Complex IV), ATPase subunit beta (Complex V), 3-hydroxybutyrate dehydrogenase (ketone body metabolism), 3-ketoacyl-acyl carrier protein reductase (fatty acid biosynthesis/quinone reductase), putative mitochondrial inner membrane protein as well as mitochondrial porin and kinesin heavy chain orthologs (Table 1). This broad representation of structural, intermediary metabolism, and OXPHOS proteins suggested that inhibition of *As*P38 MAPK significantly impacted midgut mitochondrial mass, possibly through inhibition of biogenesis. Further, given that p38 MAPK regulation of PGC-1 orthologs from invertebrates to mammals controls mitochondrial biogenesis, we hypothesized that a reduction in *A. stephensi* PGC-1 (*As*PGC-1) following inhibition of *As*P38 MAPK could account for these reductions in mitochondrial proteins. To test this, we examined expression of As*PGC-1* in mosquitoes fed *P. falciparum*-infected RBCs supplemented with 10 μM BIRB796 relative *A. stephensi* fed an identical infected blood meal supplemented with an equivalent volume of DMSO as a control. Inhibition of *As*P38 MAPK resulted in a significant decrease in As*PGC-1* gene expression relative to controls (Fig. 9), suggesting that BIRB796 reductions in midgut mitochondrial proteins in *A. stephensi* are associated with inhibition of PGC-1 expression that is analogous to that observed in other species.

### *As*P38 MAPK signaling regulates glycolysis and protein translation in *A. stephensi*

**Fig. 10:**
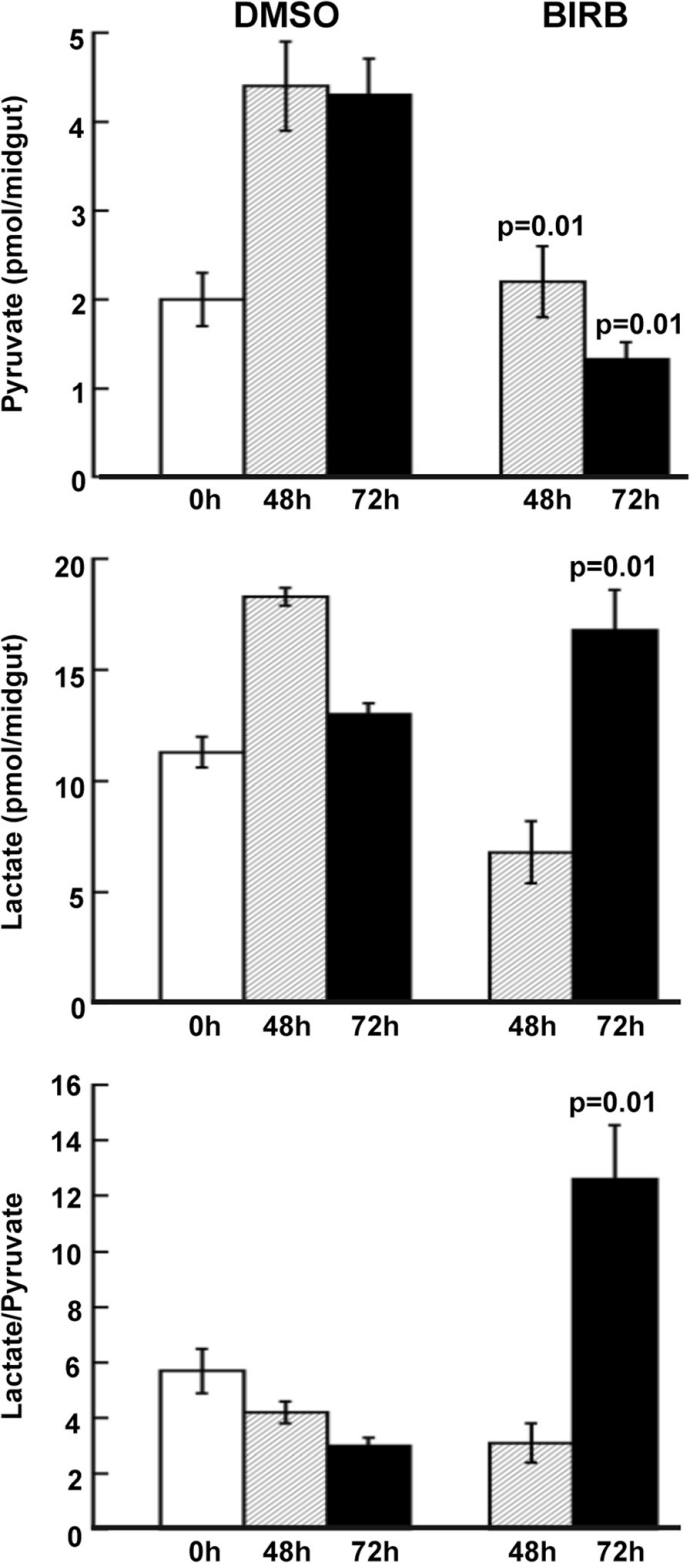
Inhibition of *As*P38 MAPK altered pyruvate and lactate concentrations in the *A. stephensi* midgut. *A. stephensi* were fed a *P. falciparum*-infected blood meal supplemented with either 10 μM BIRB796 or an equivalent volume of DMSO and 300 midguts/group were collected at 0, 48, and 72 h post-feeding to determine pyruvate and lactate levels. Graphs represent means ± SEMs of 3 independent measurements. Statistical analysis was performed by one-way ANOVA, followed by Bonferroni’s post-test for multiple comparisons between treatments (BIRB796) and controls (DMSO); significant p*-*values are shown

Significantly decreased Complex I, II, IV and V subunits suggested that *As*P38 MAPK inhibition would be associated with reduced OXPHOS. In addition to mitochondrial biogenesis, p38 MAPK signaling and activation of PGC-1α and PGC-1β have also been implicated in the control of glucose and lipid homeostasis [54, 55, 57, 56]. In particular, PGC-1α is known to drive expression of the gluconeogenic enzymes PEPCK and G6Pase in liver, while PGC-1β enhances fatty acid oxidation and glycolysis, principally through the activation of glucokinase and PK. At 24 h following provision of a *P. falciparum*-infected blood meal with 10 μM BIRB796, we noted that three proteins associated with glycolysis were under-represented following inhibition of *As*P38 MAPK (Table 1), suggesting that these enzymes are positively regulated by *As*P38 MAPK signaling. Specifically, we observed decreased levels of hexokinase, triosephosphate isomerase, and PK (Table 1). While these changes indicate an impact on glycolysis, decreased levels of nine key mitochondrial proteins (Table 1) suggest that the main effects of *As*P38 MAPK are due to modulation of mitochondrial biogenesis/function (Table 1). In support of this hypothesis, reduced glucose consumption and a shift from OXPHOS to glycolysis were observed in midguts from *A. stephensi* fed a *P. falciparum*-infected blood meal supplemented with 10 μM BIRB796 compared to an identical infected blood meal supplemented with an equivalent volume of DMSO as a control. Midgut pyruvate concentrations were decreased at 48 and 72 h by supplementation with BIRB796 (Fig. 10, top panel), whereas lactate concentrations were significantly increased at 72 h (Fig. 10, middle panel). By adding pyruvate to lactate at each time point (as an equivalent for total glucose utilization), it is clear that at 48 h there is a 1.7-fold increase in glucose consumption upon ingestion of infected blood whereas with BIRB796, no such increase occurs. A higher lactate-to-pyruvate ratio (3.8-fold of controls; Fig. 10, bottom panel) in mosquitoes fed an infectious blood meal containing BIRB796, confirms that *As*P38 MAPK is indeed involved in the regulation of midgut glucose catabolism.

Following *As*P38 MAPK inhibition, we also noted a significant under-representation of proteins involved in the processes of protein translation and synthesis such as eukaryotic initiation factor (eIF)-3E and -3I, as well a number of ribosomal and RNA-binding proteins (Table 1). Activation of p38 MAPK has been associated with increased protein translation via signaling through MK2, which enhances mRNA stability and translation [47, 90]. Therefore, the loss of midgut MK2 phosphorylation following inhibition of *As*P38 MAPK (Fig. 3) likely accounts for the observed decrease in protein translation machinery (Table 1).

Collectively, our inferences regarding proteins in Table 1 were supported by hypergeometric algorithm analysis followed by Benjamini-Hochberg correction for overrepresentation of pathways (Table 3). This analysis indicated that the most affected pathways were those associated with protein translation (ribosome, RNA transport and degradation), glucose catabolism (OXPHOS, TCA cycle, pyruvate metabolism, glycolysis), and infection (epithelial response and phagocytosis).

**Table 3:** Over-represented pathways in the *A. stephensi* midgut upon BIRB796-mediated inhibition of *As*P38 MAPK

Pathway name^a^	Pathway ID	Pathway uploaded gene count	Genes in InnateDB for this entity	Pathway p-value	Pathway p-value (corrected)	Gene symbols
Ribosome	474	17	87	2.89E-23	1.53E-22	RPL10; RPL10A; RPL13; RPL14; RPL15; RPL18; RPL23; RPL27A; RPL31; RPL4; RPL5; RPL7; RPS16; RPS19; RPS3; RPS3A; RPSA
Oxidative phosphorylation	576	4	131	6.09E-05	2.31E-04	COX7A1; NDUFB10; NDUFS1; SDHB
Glycolysis/Gluconeogenesis	414	4	64	3.51E-04	0.001	ALDH7A1; HK2; PKM2; TPI1
Metabolic pathways	4373	15	1115	4.20E-04	0.001	ALDH7A1; ANPEP; ASAH1; ATP6V1C1; ATP6V1H; HIBADH; HK2; MGAM; NDUFB10; NDUFS1; PGD; PKM2; SDHB; SUCLA2; TPI1
Galactose metabolism	407	2	27	0.009	0.021	HK2; MGAM
Citrate cycle (TCA cycle)	464	2	30	0.011	0.021	SDHB; SUCLA2
Propanoate metabolism	472	2	32	0.012	0.023	ALDH7A1; SUCLA2
Fructose and mannose metabolism	548	2	35	0.015	0.025	HK2; TPI1
Carbohydrate digestion and absorption	10389	2	41	0.020	0.031	HK2; MGAM
Pyruvate metabolism	450	2	40	0.019	0.031	ALDH7A1; PKM2
Valine, leucine and isoleucine degradation	453	2	44	0.023	0.034	ALDH7A1; HIBADH
Amino sugar and nucleotide sugar metabolism	466	2	48	0.027	0.035	CHIT1; HK2
Glutathione metabolism	534	2	48	0.027	0.035	ANPEP; PGD
Starch and sucrose metabolism	536	2	49	0.028	0.036	HK2; MGAM
Epithelial cell signaling in infection	420	2	54	0.033	0.040	ATP6V1C1; ATP6V1H
RNA transport	10361	3	149	0.044	0.052	EIF3E; EIF3I; PABPC1
RNA degradation	5710	2	68	0.050	0.056	HSPD1; PABPC1
Lysosome	4356	2	121	0.135	0.143	ASAH1; ATP6V1H
Phagosome	10394	2	145	0.180	0.187	ATP6V1C1; ATP6V1H

^a^Data from Table 1 were utilized to run a hypergeometric algorithm following a Benjamini-Hochberg correction for significance. Pathways not relevant to mosquito metabolism were deleted from the analysis

## Discussion

P38 MAPK is an evolutionarily conserved signaling protein with critical activity for innate immunity, intermediary metabolism, and mitochondrial function. In mammals, PAMP induction of Toll-like receptor (TLR) signaling activates p38 MAPK, JNK, and NF-κB-dependent transcription for the generation of distinct pro-inflammatory cytokines and cellular responses [91]. In *Drosophila*, p38 MAPK contributes to the regulation of the immune effectors through the Toll- or immune deficient (IMD)-independent pathways [19]. In *C. elegans*, vestiges of insect and mammalian Toll/TLR signaling are evident, but the nematode genome does not encode orthologs of MyD88, NF-κB, or IMD, suggesting that p38 MAPK signaling predates involvement of NF-κB in immunity [21]. However, p38 MAPK does not always positively regulate immune effector genes, but rather seems to modulate the quality and quantity of these effectors. In p38b mutant *D. melanogaster*, some genes encoding immune effectors and AMPs were up-regulated while some were down-regulated [20]. Our data suggested similar complexity in that *As*P38 MAPK positively regulated *Defensin1* gene promoter activity under LPS stimulation, negatively regulated *Cec* gene promoter activity for both LPS and PGN stimulation, and did not alter the regulation of *Gam* promoter activity under either immune stimulus in haemocyte-like ASE cells *in vitro* (Fig. 5). In the midgut epithelium, *As*P38 MAPK negatively regulated a suite of known anti-parasite effector genes to varying degrees, but positively regulated *DEF1* gene expression in the same tissue (Fig. 6). Our results are consistent with p38 MAPK regulation of *Def* in *A. aegypti* [23] and for *Cec* in *Aedes albopictus* and *D. melanogaster* [20, 25].

In addition to immunity, p38 MAPK signaling is central to metabolic regulation and redox homeostasis. In this context, the principal mediators in mammals include PGC-1α and PGC-1β. In particular, activated p38 MAPK regulates expression of PGC-1 orthologs and can also directly phosphorylate substrates to modulate protein stability and activity [92–95]. Our data showed that p38 MAPK inhibition reduced PGC-1 midgut transcript levels in *P. falciparum*-infected *A. stephensi* (Fig. 9), which together with our biochemical and proteomics data (Fig. 10, Table 1), suggest that *As*P38 MAPK-dependent regulation of PGC-1 controls midgut glucose utilization via mitochondria. In mammalian liver, PGC-1α and PGC-1β coordinately regulate gluconeogenesis and glycolysis in response to different stimuli (reviewed in [49]), and are largely redundant in the regulation of mitochondrial oxidative metabolism through coactivation of the peroxisome proliferator-activated receptors [50, 51], oestrogen-related receptors [52], and nuclear respiratory factors [53]. Intriguingly, however, the biological role of the relatively more ancient mammalian PGC-1β [95] appears to be reflected in *A. stephensi*. That is, inhibition of *As*P38 MAPK downregulated midgut glycolysis and, more so, OXPHOS as judged by a decrease in hexokinase and PK proteins at 24 h post-treatment that was followed by a decrease in pyruvate by 48 h and an increase in lactate from pyruvate by 72 h (Fig. 10). In another context, PGC-1β control of mammalian NF-κB expression and activity suggests that a similar metabolic-immune signaling axis is mediated by PGC-1 that complements mitochondrial ATP and ROS-mediated gene activation in the *A. stephensi* midgut (Fig. 8).

Inhibition of *As*P38 MAPK resulted in increased levels of midgut mitochondrial superoxide and stress-induced peroxide at 6 h following provision of BIRB796 in a saline meal (Fig. 7a, b and c) that are likely sustained through at least 24 h following *P. falciparum* infection by reduced levels of SOD2 and catalase (Table 1). This increase in steady-state concentrations of midgut ROS that cannot be mitigated by endogenous antioxidants likely contributes to reduced short-term survivorship of *A. stephensi* relative to controls (Fig. 7d). Similar observations in both mammals and in *D. melanogaster* suggest that p38 MAPK regulation of antioxidants is critical for cellular response to oxidative stress. In particular, inhibition of p38α in mouse embryonic fibroblasts led to ROS accumulation upon exposure to hydrogen peroxide, significantly increasing cell death that could be rescued with antioxidant treatment [40]. In *D. melanogaster*, p38b and p38a/b double mutants exhibited significantly decreased short-term survival to continuous feeding with hydrogen peroxide compared to wild type controls [38]. Further, the more extensive effects of shortened lifespans observed in p38b mutants and p38a/b double mutants could be rescued by overexpression of p38b [38], suggesting that p38 MAPK-dependent signaling is critical for both short-term protection against oxidative stress and longer term survival. Intriguingly, PGC-1α is required for the induction of antioxidants (SOD1, SOD2, catalase, GPx1) to suppress ROS levels in mice *in vivo* and in murine neuronal cells *in vitro* [96], suggesting that a lack of PGC-1 activity in fruit flies and mosquitoes could also explain the effects of p38 MAPK inhibition on sensitivity to oxidative stress.

Bacteria present in the midgut have been shown to modulate the mosquito immune response through activation of basal immunity and in the absence of midgut flora *Anopheles* mosquitoes are more susceptible to *Plasmodium* infection [97]. Although blood feeding leads to a bloom of gut microflora in *Anopheles* [98–100], it also concurrently reduces the diversity of the gut bacterial community dramatically [100]. Therefore, while it is likely that midgut bacteria contribute to p38 MAPK activation during blood feeding, our data suggest that this effect is altered by the presence of malaria parasites. In particular, we have shown that there are no significant differences in midgut bacteria CFUs in mosquitoes fed uninfected versus *Plasmodium*-infected blood either at 24 or 48 h post feeding (Additional file 2). Therefore, we can infer that the contribution of bacteria and endotoxin to the observed changes in *As*P38 MAPK signaling are consistent between mosquitoes fed uninfected and infected blood. As such, any differences observed in mosquitoes fed an infectious blood meal are due to the presence of parasites and not to quantitative alterations in midgut flora. Interestingly, infected RBCs have been shown to contain endotoxin-like lipidic molecules [101] and could, therefore, activate p38 MAPK signaling via pathways that are similar to those activated by bacterial endotoxin.

In mammals, p38 MAPK activation has been associated with increased mRNA translation via phosphorylation of MK2, which enhances mRNA stability and translation [47, 90]. Inhibition of *As*P38 MAPK was associated with reduced phosphorylation of MK2 in ASE cells and in the midgut epithelium (Fig. 3) and with significantly reduced levels of proteins associated with mRNA translation in the same tissue at 24 h post-treatment (Table 1). A previous study reported significantly enhanced translation of midgut mRNAs during the first 22-26 h following *P. falciparum* infection in *A. gambiae* with notably few changes in mRNAs for immune genes [102], suggesting that parasite-induced activation of p38 MAPK signaling in the midgut epithelium of both important malaria vectors regulates a conserved response to infection. Alternatively, compromised ATP status emerging from lower glucose catabolism can also decrease the high-energy consuming process of synthesizing ribosomal RNAs. Based on data from Fig. 10, the ATP generated following treatment with BIRB796 can be estimated as 44 % of controls, conditions akin to starvation that may also decrease protein synthesis as evidenced by the 5-fold ratio of underrepresented/overrepresented proteins (Table 1).

## Conclusions

**Fig. 11:**
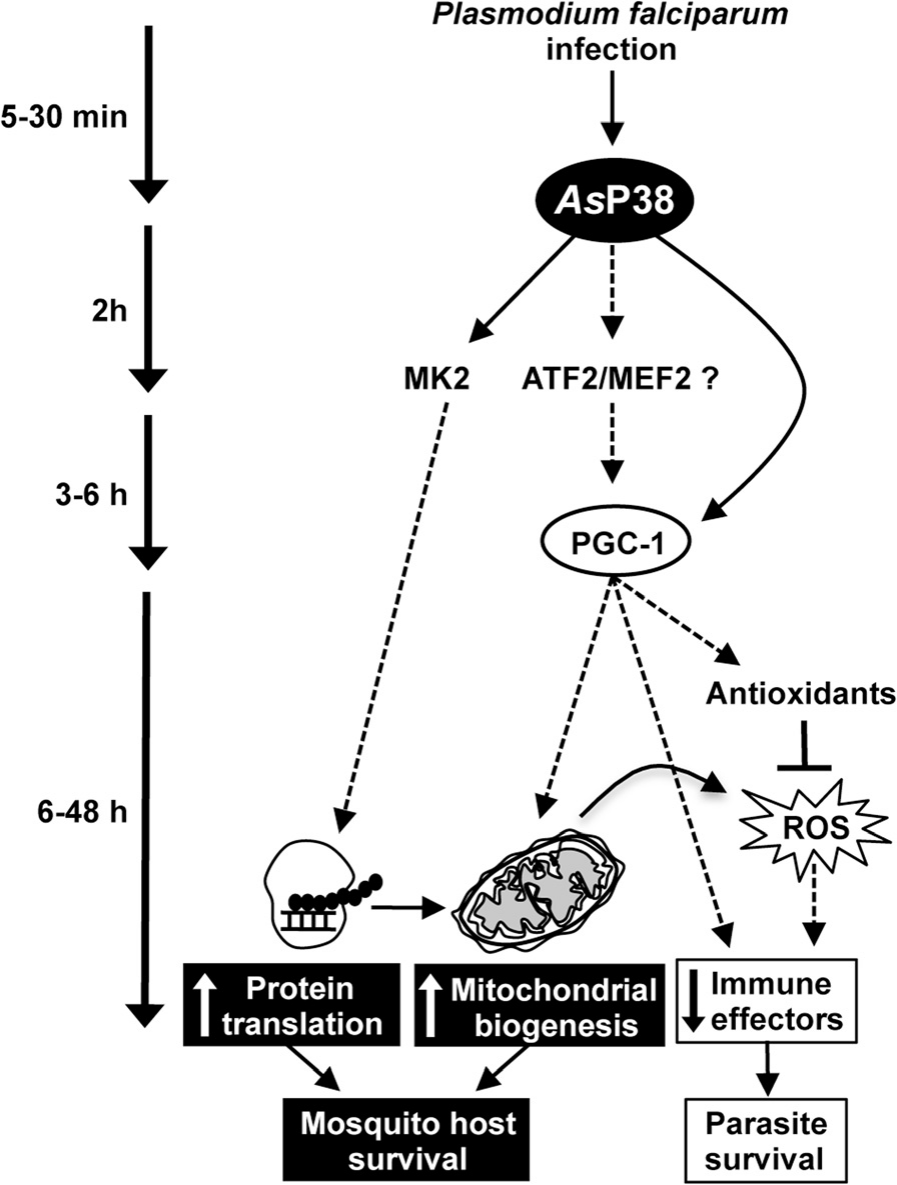
Model for *As*P38 MAPK driven tolerance to *Plasmodium* infection. Malaria parasite infection in *A. stephensi* increases *As*P38 phosphorylation in the midgut epithelium leading to the activation of transcription factors that drive expression of proteins involved in glycolysis, mitochondrial biogenesis, and protein translation, which results in enhanced energy efficiency and protein synthesis. Activation of *As*P38 enhances the synthesis of antioxidant enzymes, which reduce damage due to oxidative stress, but which also contribute to decreased ROS-mediated immune gene activation. Together, these processes increase mosquito host survival in the context of parasite development and transmission

In this study, we used small molecule inhibitors as tools to probe the significance of parasite infection-induced *As*P38 MAPK phosphorylation and infer that this activation induces PGC-1 expression, which contributes to increased glycolysis, mitochondrial biogenesis, and synthesis of antioxidant enzymes. Further, *As*P38 MAPK activation enhances MK2 phosphorylation and, as interpreted from BIRB796 inhibition, mRNA translation in the midgut epithelium, events that are likely to be functionally connected. Hence, parasite-mediated activation of p38 MAPK signaling in the *A. stephensi* midgut likely protects the host from oxidative stress and enhances survivorship, but also decreases the activation of energetically costly anti-parasite gene expression. Increased glycolysis, OXPHOS and mRNA translation can mitigate energy loss and cellular damage due to infection (Fig. 11), suggesting that *As*P38 MAPK signaling contributes to mosquito survival during *P. falciparum* infection.

Our data have revealed that p38 MAPK activation during *P. falciparum* infection of *A. stephensi* is associated with enhanced fitness – as defined by increased protein synthesis, mitochondrial biogenesis, and resistance to oxidative stress– and a reduced innate immune response to infection. Given that insecticide resistance has been associated with enhanced mitochondrial metabolism (Complexes I and IV; [103]) and antioxidant levels (GST, TPX2; [103, 104]), genetic or pharmacological strategies to inhibit p38 MAPK signaling in natural populations of mosquitoes could decrease malaria parasite infection prevalence *and* enhance acute susceptibility to chemical pesticides. Together with our previous work [63], these data provide additional evidence for networked regulation of mitochondrial metabolism, antioxidant response and immunity in *A. stephensi*, which can be leveraged for improved methods for malaria transmission control.

## Additional files

Additional file 1:
***P. falciparum*** **parasite products (** ***Pf*** **Ps) induced A** ***s*** **P38 MAPK phosphorylation** ***in vitro*** **relative to control.** (TIFF 1850 kb) Additional file 2:
***P. falciparum*** **-infected RBCs did not significantly alter midgut bacterial growth relative to uninfected RBCs.** (TIFF 2079 kb)

## Abbreviations

ASE: *A. stephensi* embryonic
*As*P38: *A. stephensi* p38
BLAST: Basic Local Alignment Search Tool
ERK: Extracellular signal-regulated kinase
JNK: c-Jun N-terminal kinases
G6Pase: glucose-6-phosphatase
FTPP: Freeze-thawed *P. falciparum* products
IMD: Immune deficient
IIS: Insulin/insulin-like growth factor signaling
TOR: Insulin-target of rapamycin
IFN-γ: Interferon
LC-MS/MS: Liquid chromatography/tandem mass spectrometry
MAPK: Mitogen-activated protein kinase
MK2: MAPK-activated kinase 2
NF-κB: Nuclear factor
OXPHOS: Oxidative phosphorylation
PAMPs: Pathogen-associated molecular patterns
PGC-1α: Peroxisome proliferator-activated receptor gamma coactivator-1α
PGN: Peptidoglycan
PEPCK: Phosphoenolpyruvate carboxykinase
PK: Pyruvate kinase
RNOS: Reactive nitrogen and oxygen species
(ROS): Reactive oxygen species
RBCs: Red blood cells
TLR: Toll-like receptor
STAT-1: Signal transducer and activator of transcription
(SOD2): Superoxide dismutase
