# Supplementary figures and images for: Anopheles stephensi p38 MAPK signaling regulates innate immunity and bioenergetics during Plasmodium falciparum infection
Source: Parasit Vectors. 2015 Aug 19;8:424. doi: 10.1186/s13071-015-1016-x (PMC4539710; doi:10.1186/s13071-015-1016-x)

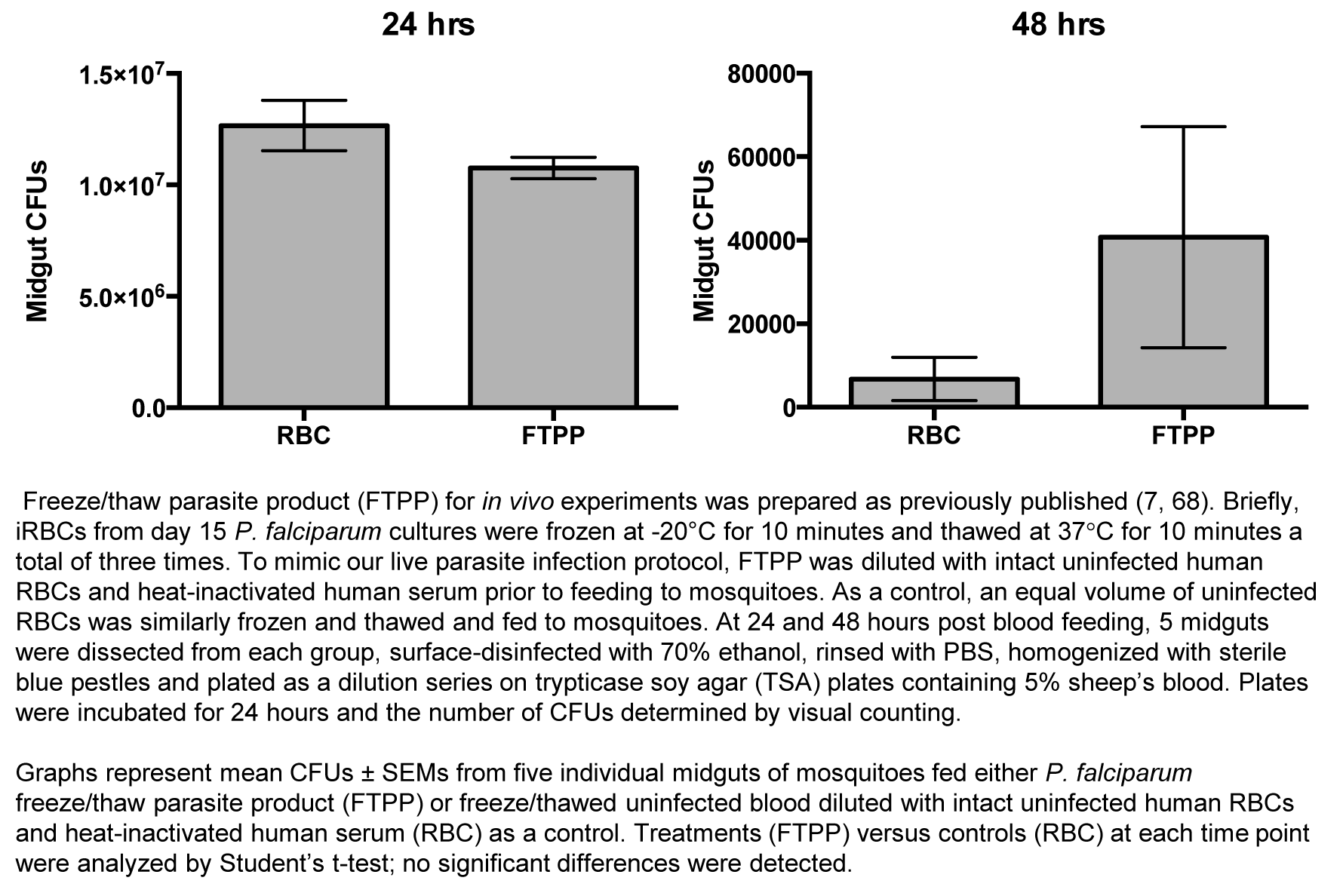

Supplement: Additional file 1: — P. falciparum parasite products ( Pf Ps) induced A s P38 MAPK phosphorylation in vitro relative to control. (TIFF 1850 kb) [file 13071_2015_1016_MOESM1_ESM.tif]

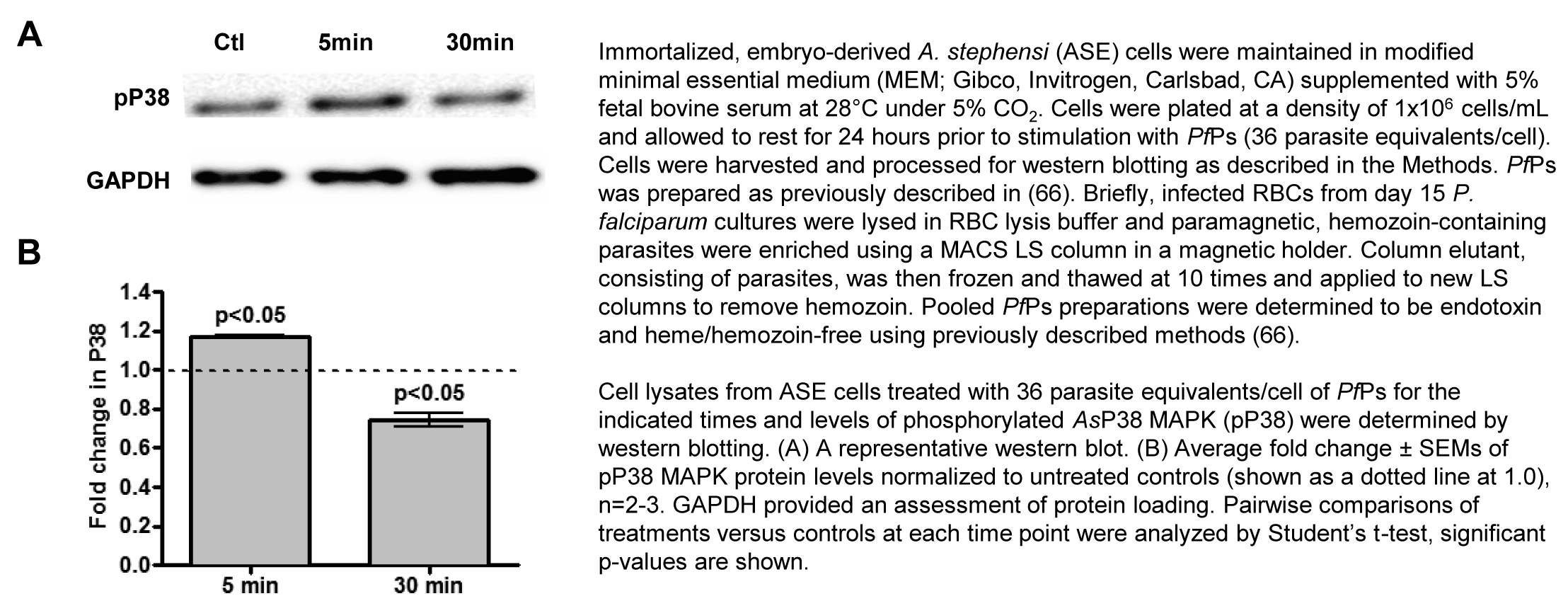

Supplement: Additional file 2: — P. falciparum -infected RBCs did not significantly alter midgut bacterial growth relative to uninfected RBCs. (TIFF 2079 kb) [file 13071_2015_1016_MOESM2_ESM.tif]
